# Supplementary material for: Anticipated health behaviour changes and perceived control in response to disclosure of genetic risk of breast and ovarian cancer: a quantitative survey study among women in the UK
Source: BMJ Open. 2017 Dec 22;7(12):e017675. doi: 10.1136/bmjopen-2017-017675 (PMC5770899; doi:10.1136/bmjopen-2017-017675)
Supplement: Supplementary file 1 [file bmjopen-2017-017675supp001.pdf]

Supplementary Table 1. Full set of responses for outcome variables

If I knew my risk of breast and ovarian cancer…	N (%)
**I would try harder to have a healthy lifestyle**	
Strongly disagree	33 (3.9)
Disagree	50 (6.0)
Neither agree/disagree	143 (17.1)
Agree	425 (50.8)
Strongly agree	186 (22.2)
**It would give me more control over my life**	
Strongly disagree	22 (2.6)
Disagree	135 (16.1)
Neither agree/disagree	228 (27.2)
Agree	387 (46.2)
Strongly agree	65 (7.8)
**I would feel it wasn't worth making lifestyle changes**	
Strongly disagree	169 (20.2)
Disagree	397 (47.4)
Neither agree/disagree	152 (18.2)
Agree	104 (12.4)
Strongly agree	15 (1.8)
**I would feel less free to make choices in my life**	
Strongly disagree	112 (13.4)
Disagree	402 (48.0)
Neither agree/disagree	180 (21.5)
Agree	129 (15.4)
Strongly agree	14 (1.7)
