# Supplementary material for: Anticipated health behaviour changes and perceived control in response to disclosure of genetic risk of breast and ovarian cancer: a quantitative survey study among women in the UK
Source: BMJ Open. 2017 Dec 22;7(12):e017675. doi: 10.1136/bmjopen-2017-017675 (PMC5770899; doi:10.1136/bmjopen-2017-017675)
Supplement: Author's manuscript [file bmjopen-2017-017675.draft_revisions.pdf]

BMJ Open

BMJ Open is committed to open peer review. As part of this commitment we make the peer review history of every article we publish publicly available.

When an article is published we post the peer reviewers' comments and the authors' responses online. We also post the versions of the paper that were used during peer review. These are the versions that the peer review comments apply to.

The versions of the paper that follow are the versions that were submitted during the peer review process. They are not the versions of record or the final published versions. They should not be cited or distributed as the published version of this manuscript.

BMJ Open is an open access journal and the full, final, typeset and author-corrected version of record of the manuscript is available on our site with no access controls, subscription charges or pay-per-view fees (http://bmjopen.bmj.com).

If you have any questions on BMJ Open's open peer review process please email editorial.bmjopen@bmj.com

BMJ Open

Anticipated response to disclosure of genetic risk of breast and ovarian cancer: a survey study among women in the UK

Journal:	*BMJ Open*
Manuscript ID	bmjopen-2017-017675
Article Type:	Research
Date Submitted by the Author:	08-May-2017
Complete List of Authors:	Meisel, Susanne; Kings College London<br>Fraser, Lindsay; Institute for Women's Health, Department of Women's Cancer<br>Side, Lucy; University College London Institute for Women's Health<br>Gessler, Sue; University College London Hospitals NHS Foundation Trust, ; UCL Institute for Women's Health and NIHR University College London Hospitals Biomedical Research Centre, Department of Women's Cancer<br>Hann, Katie; University College London Institute for Women's Health, Department of Women's Cancer<br>Wardle, Jane; University College London, Dept. of Epidemiol<br>Lanceley, Anne; UCL, Department of Women's Cancer
**Primary Subject Heading**:	Genetics and genomics
Secondary Subject Heading:	Obstetrics and gynaecology
Keywords:	Stratification, Breast cancer, Ovarian cancer, Predictive genetic testing, Risk, Implementation

SCHOLARONE™
Manuscripts

For peer review only

For peer review only - http://bmjopen.bmj.com/site/about/guidelines.xhtml

TITLE: Anticipated response to disclosure of genetic risk of breast and ovarian cancer: a survey study among women in the UK

Authors: Susanne F Meisel PhD[1], Lindsay Fraser BSc[2], Lucy Side MD[2], Sue Gessler PhD[2], Katie E J Hann MSc[2], Jane Wardle PhD[3]† and Anne Lanceley PhD[2] for the PROMISE study team

[1]Institute of Psychiatry, Psychology and Neuroscience, King's College London, London, UK.

[2]Department of Women's Cancer, EGA UCL Institute for Women's Health, University College London, London, UK.

[3]Department of Behavioural Science and Health, Institute of Epidemiology and Health Care, University College London, London, UK.

Corresponding Author:

Anne Lanceley, PhD

237c

Medical School Building

74 Huntley Street

London, WC1E 6AU

Email: a.lanceley@ucl.ac.uk

Phone: +4420 7679 6807

For peer review only

Abstract

Background: Genetic risk assessment for breast and ovarian cancer (BCOC) is expected to make major inroads into mainstream clinical practice. However, there are concerns in professional circles that predictive genetic testing without adequate support could lead to adverse psychological and behavioural responses to risk disclosure.

Objective: To examine anticipated responses to disclosure of genetic risk for BCOC, and establish demographic and personal correlates of adverse anticipated responses in a population-based sample of women.

Design: Cross-sectional survey study carried out by the UK Office for National Statistics.

Setting: Face-to-face computer-assisted interviews conducted by trained researchers in participants' homes.

Participants: 837 women randomly chosen from households across the UK identified from the Royal Mail's Postcode Address File.

Outcome measures: Anticipated response to disclosure of BCOC risk.

Results: Very few women (2%) thought that it would 'never' be a good time to get a genetic test for risk of BCOC. In response to a genetic test result, most women (72%) indicated 'I would try harder to have a healthy lifestyle' and over half (55%) felt 'it would give me more control over my life'. These effects were independent of demographic factors or perceived risk of BCOC in Bonferroni-corrected multivariate analyses. However, a minority of women (14%) felt it 'isn't worth making lifestyle changes', and that 'I would feel less free to make choices in my life' (16%) in response to BCOC risk disclosure. The former belief was more likely to be held by women who were educated below university degree level ($p < 0.001$) after adjusting for other demographic and personal correlates.

For peer review only

Conclusion: These findings indicate that women in the UK largely anticipate that they would respond positively to BCOC risk disclosure. However, those with a lower than degree level education may be more likely to take a deterministic approach to genetic testing.

Key words: Stratification, Breast cancer, Ovarian cancer, Predictive genetic testing, Risk, Implementation

Strengths and limitations of this study

- Stratified random sampling, the 'gold-standard' for survey recruitment, was used in this study.
- The study reports on anticipated responses to genetic BCOC risk information from a large general population sample of women living in the UK.
- The study findings are based on a hypothetical scenario rather than anticipated responses to BCOC risk information of individuals who were actually offered genetic testing.
- A general hypothetical scenario about receiving genetic BCOC risk information was used instead of asking participants to consider a hypothetical scenario of receiving a high or low risk genetic test result.

For peer review only

Source of funding:

This research was funded by grants from The Eve Appeal (London, UK, grant code: 509050) and Cancer Research UK (London, UK; grant code: 508007). Jane Wardle was supported by Cancer Research UK as part of the CRUK-UCL Cancer Centre.

Conflicts of interest:

All authors have completed the ICMJE uniform disclosure form at www.icmje.org/coi_disclosure.pdf and declare: SFM and KEJH had financial support from Cancer Research UK and the Eve Appeal for the submitted work; no financial relationships with any organisations that might have an interest in the submitted work in the previous three years; no other relationships or activities that could appear to have influenced the submitted work.

Authors contributions:

SFM participated in study design, analysed the data and drafted the manuscript; KEJH helped with data interpretation and critically revised the manuscript; LS, LF, SG, AL and JW conceived of the study, participated in study design and coordination and critically revised the manuscript. All authors have seen and approved the final manuscript.

Data sharing statement

The data is available on request from the corresponding author: a.lanceley@ucl.ac.uk

Word count: 3827

Number of tables: 3

For peer review only

Introduction

Advances in genetic technologies have led to the identification of gene mutations associated with an increased risk of developing breast and ovarian cancer (BCOC). In addition to germline mutations in BRCA 1 and BRCA 2, which confer a very high lifetime risk of BCOC (39%- 65% and 11%-37%, respectively) [1-3], a growing list of moderate- and lower-risk markers specific to each cancer type have been identified [4-7]. These influence cancer development in a larger proportion of the population despite small effect sizes. It is anticipated that, in future, some moderate risk markers will be included in existing panel tests used in clinical practice [8-10].

To date, genetic testing of BRCA1 and BRCA2 is conducted in clinical practice only, in individuals with a strong family history, or after a cancer diagnosis. However, it has been argued that this approach is likely to miss a substantial proportion of women at high risk (~30%), particularly if the family is small, the mutation was transmitted through the paternal line, or the individual has a number of lower risk mutations whose effects are additive [11-14]. Given that genetic testing for BCOC risk can now be carried out at a relatively low cost, there have been calls to make it available on a population level, based on the premise that timely awareness of genetic risk would empower individuals to make informed decisions about cancer risk management, allow for early intervention, and ultimately, improve clinical outcomes [15]. In fact, genetic testing for BCOC susceptibility can already be purchased over the Internet (www.23andme.com; www.getcolor.com). The notion that individuals have a 'right to know' their genetic information has also gained traction in recent years [16-19].

However, concerns persist about patient safety related to poor psychological adjustment following genetic risk disclosure. Studies investigating reactions to disclosure of BRCA status commonly do not find evidence for persistent negative psychological outcomes beyond one year after result disclosure [20-

For peer review only

[23]. There is also some evidence suggesting that genetic test results indicating a higher risk of disease can lead to behaviour change such as uptake of breast and colorectal cancer screening [20 24 25], although two systematic reviews found no significant impact of genetic risk information on other behaviours [26 27] including smoking, physical activity, use of medication or vitamins, diet, alcohol use and use of sunscreen. However, genetic testing has hitherto only been performed in women with a strong family history of BCOC, so these results may not generalise to a population largely unaware of any increased risk.

Currently, one-to-one genetic counselling is strongly embedded within genetic testing services, offering education and support throughout the process. If genetic testing were introduced on a population level, the current model of delivering genetic counselling would no longer be feasible. It has been argued that returning genetic risk test results for highly debilitating conditions such as BCOC without appropriate professional guidance would leave room for misinterpretation and uncertainty, increasing the risk of adverse psychological outcomes [28-31]. In recent years, research has begun to investigate new approaches of providing genetic services. Mainstreaming genetic testing, by offering women with a diagnosis of ovarian cancer testing directly through the oncology clinic and providing post-test genetic counselling via clinical genetics for those with a hereditary gene mutation, is one example. Research has found that mainstreaming genetic testing in this way can successfully reduce time and resources spent on the processes [32] and does not appear to negatively affect patients' psychological well-being [33]. Genetic counselling with patients and healthy family members via telephone or video consultations instead of face-to-face genetic counselling has also been found to be feasible and patients are generally satisfied [34-36]. These new approaches to offering genetic testing and providing genetic counselling for cancer susceptibility may be advantageous in future if testing is offered to patients more widely.

For peer review only

Although a wealth of studies have investigated anticipated psychological reactions to disclosure of BCOC genetic risk in higher risk groups [20 37 38] little is known about this issue in women unselected for family history. One qualitative study found that women in the UK would support the idea of genetic testing for risk of ovarian cancer because they expected it to spark positive lifestyle changes and provide a means of control over cancer development [39]. However, in a similar study investigating attitudes towards genetic testing for breast cancer risk in the Netherlands, women were more ambivalent about disclosure of genetic risk; although there was recognition of the benefits of testing, some women were uncertain about their reactions to risk disclosure and the impact it would have on their lives [40].

An early population-based survey investigated attitudes towards genetic testing for breast cancer among 836 women living in Washington State [41]. Results showed that over three quarters of the sample expected that awareness of their genetic risk of breast cancer would give them more control over their life, and a similar number reported that they would 'definitely' or 'probably' take part in testing if it was offered to them. A more recent population-based study with over 800 participants in the Netherlands compared attitudes towards genetic testing between 2002 and 2010 [42]. Results showed that positive expectations about the utility of genetic testing had increased over the 8-year interval. However, about a third of participants believed that awareness of genetic risk would deprive people of the freedom to live as they want, and this proportion remained unchanged over time. Although these findings give an indication about the acceptability of population-based genetic testing, they may be culture-specific and may therefore not generalise to other populations. Furthermore, the latter survey asked only about attitudes towards genetic testing more broadly; attitudes to genetic risk disclosure specific to BCOC may be different.

For peer review only

The aim of the present study was to examine the anticipated response to disclosure of genetic risk for BCOC and to explore demographic and personal correlates of anticipated response among a population sample of women in the UK. Identifying subgroups who are more likely to anticipate a negative response to learning about their genetic risk of developing these cancers, before genetic testing is integrated into mainstream healthcare (e.g. by including it in current cancer screening programmes), gives scope for timely intervention to avoid further increasing disparities in healthcare.

Methods and Procedure

Sample

Data were collected by the UK Office for National Statistics (ONS) in their monthly survey which is conducted on behalf of a range of organisations (e.g. government departments, non-governmental institutions and academic departments). Each month, 2010 households are identified from the Royal Mail's Postcode Address File using stratified random probability sampling. Selected addresses are contacted up to eight times at different times and days of the week to maximize response rates. One person aged over 16 from each household is randomly chosen to complete a computer-assisted face-to-face interview with trained researchers. Surveys commonly include a variety of different topics, making participation bias unlikely; although participants can opt out at any point. Questions on the 'genetics and screening' module were included in data collection waves in January and March 2014.

Measures

The 'genes and cancer' module was introduced with a short statement: '*Genes contain the 'instruction manual' of life, called DNA. Genes are passed from parents to their children. Nowadays, it is possible to predict whether someone is likely to develop certain diseases by looking at their genes. This is called genetic testing'*. No further information was given, as we were interested in women's attitudes towards

For peer review only

BCOC genetic testing based on their current understanding of it. Participants were asked several questions in relation to genetic testing for BCOC risk and attitudes on breast cancer screening, some of the findings have previously been published [43].

Outcome variables

We asked 'When do you feel is the best time for a person to have a genetic test for breast cancer and ovarian cancer?', with response options of 'just after birth', 'during school', 'just before marriage', 'before they plan to have a child', 'when they are unwell', 'never', and 'unsure' to gauge whether women were generally supportive about the idea of genetic testing. This question was adapted from Durfy and colleagues [44].

Positive anticipated responses to disclosure of genetic risk for breast and ovarian cancer was assessed with two statements: 'If I knew my genetic risk of breast cancer and ovarian cancer I would try harder to have a healthy lifestyle', 'If I knew my genetic risk of breast cancer and ovarian cancer, it would give me more control over my life'. Attitudes reflecting a more deterministic view were also assessed with two statements: 'If I knew my genetic risk of breast cancer and ovarian cancer, I would feel it wasn't worth making lifestyle changes', and 'If I knew my genetic risk of breast cancer and ovarian cancer, I would feel less free to make choices in my life'. All items were scored on a five-point Likert scale ranging from 'strongly disagree'- 'strongly agree', and were adapted from previous research [41 42].

Predictor variables

Perceived risk of BCOC was each assessed with one question 'Compared with other women of your age, what do you think are your chances of getting breast [ovarian] cancer' with response options of: 'much lower than others', 'lower than others', 'the same as others', 'higher than others', 'much higher than

For peer review only

others'. For some analyses, perceived risk was grouped into three categories: 'much lower/lower than others; 'same as others', and 'higher/much higher than others'.

Age, ethnicity, educational attainment and marital status were derived using standard survey items (http://www.ons.gov.uk/ons/%20ons/index.html). Age was coded as ≤50 vs. >50 years to investigate any generational differences in anticipated reactions and attitudes. Ethnicity was classified as 'White' vs. 'ethnic minority' because the individual ethnic minority sub-groups were small. Educational attainment was classified as university degree or equivalent vs. below university degree level. Marital status was coded as married/cohabiting vs. single/widowed/divorced. Family history of any cancer was assessed with one question: 'Have your mother, father, or any of your brothers or sisters, been diagnosed with cancer?' with response options being 'yes', 'no', and 'don't know'.

Statistical analyses

We included women aged 18-74 to reflect the population to whom genetic testing for risk of BCOC would likely become available in the future. Statistical analyses were carried out using the Statistical Package for Social Sciences (SPSS) version 20.0 (SPSS Inc., Chicago, IL). We explored demographic (age, ethnicity, education) and personal predictors (marital status, family history of cancer, perceived risk of ovarian cancer, perceived risk of breast cancer) for each outcome variable using χ^2 tests. Multivariate logistic regression models were used to investigate the demographic and personal correlates of anticipated reactions to disclosure of personal BCOC risk. Bonferroni corrections were employed in all multivariate analyses to correct for multiple testing, with $\alpha = 0.0125$.

For peer review only

Results

Demographic and personal characteristics

Data for the current study were collected in two waves: January and March 2014. In the January wave, 8% (n = 166) of the 2,010 selected households were not eligible because they were businesses or empty properties. Of 1,844 eligible households, 9% (n = 171) could not be contacted and 33% (n = 608) declined to take part in the ONS survey. In the March wave, 1,853 households were eligible (92%). Of those, 13% (n = 237) could not be contacted and 31% (n = 578) chose not to take part. Therefore, the overall response rate was 57%, comparable to previous ONS surveys (ONS 2013; ONS 2012; http://www.ons.gov.uk/ons/index.html). The total female sample was n=1,095.

After excluding participants with incomplete data for any of the outcome variables (n= 120), or who were aged younger than 18 or older than 74 years (n = 138), the final sample for analysis consisted of 837 women. Table 1 presents the demographic and personal characteristics of the sample. In common with other survey studies, education was higher in the sample than in the general population; other demographic characteristics were comparable to the UK population of women aged 18-74 (ONS Census 2011: (http://www.ons.gov.uk/ons/guide-method/census/2011/index).

Although 36.4% (n = 305) of the sample reported a family history of any cancer, most women felt that they were not particularly at risk of breast or ovarian cancer; only 9.9% (n = 83) thought they were at ‘higher’ or ‘much higher’ than average risk of breast cancer, and even fewer (7.6%, n = 64) thought they were at ‘higher’ or ‘much higher’ risk of ovarian cancer.

Support of genetic testing

For peer review only

Overall, women were supportive of the idea of genetic testing for breast and ovarian cancer. Only 2.0% (n = 18) thought that it was 'never' a good time to have a genetic test. However, about a third of the sample (31.4%, n = 290) were 'unsure'. Opinions were divided on when the best time would be to get tested, with 8.3% (n = 77) thinking that it would be 'just after birth', just over a quarter (27.4%, n = 253) thought 'during school', 10.6% (n = 98) reported 'just before marriage', 16.9% (n=156) 'before they plan to have a child', and 3.4% (n = 31) thought the best time would be 'when they are unwell'. There were no demographic or personal correlates with 'ever' vs. 'never' supporting genetic testing for breast and ovarian cancer (data not shown).

Anticipated response to disclosure of genetic risk of breast and ovarian cancer

Overall, women anticipated to respond positively to awareness of their genetic risk. Most (71.9%, n = 669) said that they 'would try harder to have a healthy lifestyle', and over half (54.7%, n = 506) anticipated that it would give them 'more control over my life'. However, a minority had a more deterministic attitude, reporting that it would 'not be worth making lifestyle changes' (14.1%, n = 130) in response to genetic risk disclosure, and that they would feel 'less free to make choices in my life' (16.4%, n = 152).

In univariate analyses, there were no differences across demographic and personal variables in the expectation that disclosure of genetic risk of BCOC would lead to increased personal efforts 'to have a healthy lifestyle' (Table 2). Women who had fewer formal years of education (p = 0.041), were married (p = 0.014), or had a family history of cancer (p = 0.004) were significantly more likely to report that awareness of genetic risk would give them more control over their life (Table 2). However, none of these effects survived adjustment for covariates and multiple testing (Table 3).

For peer review only

Women with fewer years of formal education were also significantly more likely to hold a more deterministic attitude than women who had stayed in education for longer and agree that 'it wouldn't be worth making lifestyle changes' in response to genetic risk disclosure ($p = 0.001$), and this finding was maintained in multivariate analyses (OR = 1.99, 95% CI = 1.19-3.35, $p = 0.009$). Women from ethnic minority backgrounds ($p = 0.033$), those who perceived themselves at a (much) lower or (much) higher risk of breast cancer ($p = 0.023$) or ovarian cancer ($p<0.001$) were more likely to report that awareness of genetic risk would make them 'feel less free to make choices in their life', but none of these findings was maintained after adjusting for demographic and personal factors, and for multiple testing (Table 3).

Discussion

This study investigated demographic and personal correlates of anticipated responses towards disclosure of genetic risk of BCOC in a population-based sample of women in the UK. Overall, women were supportive of the idea of genetic testing for breast and ovarian cancer, with very few (2%) reporting that it would 'never' be a good time to have a genetic test. This finding matches those from earlier studies, including those from other countries, which have consistently shown that women are on the whole enthusiastic about the prospect of genetic testing for breast and ovarian cancer [39 43 45 46].

Interestingly, there was no consensus among women when the best time for a genetic test would be; relatively few women (9%) thought that it would be best to test children 'just after birth', and about a third of the sample were 'unsure'. Given the debate whether whole genome sequencing should be included into routine new-born screening in professional circles [47 48], which would by definition include testing for BRCA and other genes related to breast and ovarian cancer, it is interesting to observe that women in the UK do not seem to hold particularly strong views about this issue. However, in this study,

For peer review only

we did not give any information on current availability of genetic testing for either children or adults. It is possible that many women were unaware of current practices, including the current debate about the expansion of new-born screening. In addition, we only used a single item to assess perceptions about timing of genetic testing. Future research could investigate this area further, particularly, since early studies in the US using samples of parents indicate that interest in this technology may be high [49].

Although our results suggested that most women did not expect to be negatively affected by knowing their genetic risk status for BCOC, women who had fewer years of formal education were nearly twice more likely to believe that they would react with a deterministic attitude than those who had stayed in formal education for longer. This finding supports some earlier studies into genetic determinism [50]; although not all studies have found this link [41 51].

However, it also echoes studies that investigated attitudes towards cancer screening, which have found that women with lower socio-economic status, which is linked to educational attainment [52], were more likely to hold fatalistic attitudes towards cancer development. Fatalistic attitudes have in turn been implicated in lower levels of engagement with cancer screening [53 54]. It is possible that women who do not believe in the curability of cancer *per se* are also more likely to report that ‘it wouldn’t be worth making lifestyle changes’ in response to disclosure of BCOC genetic risk, but because we did not assess beliefs about curability of BCOC, this hypothesis remains speculative. Furthermore, research has shown that those with fewer years in education are less aware of cancer risk factors such as poor diet and physical inactivity [55]; a lack of awareness that lifestyle effects a person’s risk of cancer would also help to explain why some participants may feel that ‘it wouldn’t be worth making lifestyle changes’. Given that there are already disparities in engagement with available means of early detection for cancer (e.g. screening) across the population, with lower SES groups less likely to engage [56], it will be vital to provide

For peer review only

adequate education and information about the nature of genetic testing before it is introduced into mainstream healthcare. This point has also been highlighted by other commentators in the field [57 58]. In addition, it is important that the public has a multifaceted view of cancer and are aware of genetic and non-genetic risk factors and ways to reduce risk despite genetic test results.

Although previous studies in both higher-risk [59 60] and population samples [39 61] have shown that younger women are generally more positive towards genetic testing, we found no age differences in anticipated reactions towards BCOC genetic risk disclosure. It is interesting to speculate about the impact on our study respondents about the benefits of genetic testing as a result of the spike in media coverage that took place shortly before the survey responses were collected at the beginning of 2014. The well-known actress Angelina Jolie publicly announced her reasons for undergoing BRCA genetic testing and talked about how the knowledge that she carried a gene mutation had enabled her to choose risk-reducing surgery (double mastectomy) to manage her increased risk of developing breast cancer. This celebrity revelation may have raised awareness of the potential benefits of breast (and ovarian) cancer genetic testing across all age groups [62 63], which may at least partially account for the finding that older women were as positive about genetic testing as younger women.

We found no association between participants' anticipated response to genetic risk disclosure and perceived risk of either breast or ovarian cancer. However, since few women considered themselves to be at 'higher' or 'much higher' than average risk of BCOC, sample sizes may have been too small to detect associations. Alternatively, these findings may suggest that UK women may have a more realistic understanding of their personal risk of BCOC as reported in previous studies from other countries [64 65]. Given that all survey items were closed questions, we could not investigate this finding in more depth, but this could be done in future research.

For peer review only

The strengths of this study are the approach to sampling (the sample was selected using stratified random sampling which is the gold-standard of survey recruitment and therefore, self-selection is unlikely to have biased current findings) and the statistical adjustment for multiple testing, which gives confidence in the robustness of the findings. However, the study also had important limitations. We only assessed anticipated responses to disclosure of genetic risk of BCOC in general, not whether these differed in response to a hypothetical 'higher' or 'lower' genetic risk. All questions have been used in previous research with different samples, with comparable findings. Further research could explore whether attitudes to genetic risk disclosure of BCOC differ when considering different levels of risk. Secondly, because of survey constraints, we focused our investigation on a limited set of potential enablers and barriers of genetic testing, namely perceived benefits and deterministic attitudes.

Naturally, responses to 'real' disclosure of genetic risk information may be less positive than our results suggest; whilst the majority of participants in this study indicated anticipated effort to 'have a healthy lifestyle', research has shown that there is generally a 'gap' between intentions and actual behaviour [66]. In addition, given that many individuals have difficulty in accurately anticipating emotional reactions to life events [67 68], these findings can only give a broad indication about potential effects of disclosure of BCOC genetic risk. Since none of the questions were open ended, we could not explore the origins of negative responses in more detail, but this could be done in future research.

Women in the UK are generally positive about the prospect of population based genetic testing for risk of BCOC. However, those with fewer years in education may be less positive. Given that there are already existing disparities in uptake of available means of early detection for breast cancer, careful planning is needed to avoid increasing these further. Although the risk of psychological harm may in

For peer review only

part be mitigated by self-selection into genetic testing [69], it will be vital to understand the origins of anticipated negative responses and provide adequate education and information about the nature of genetic testing before its integration into mainstream healthcare. This study provides evidence to suggest that before population genetic testing is made available, education is needed that is inclusive of people with different levels of information literacy. Further studies will be needed to evaluate education approaches and their impact on women's choices about whether to participate.

For peer review only

Reference List

1. Antoniou A, Easton DF. Average risks of breast and ovarian cancer associated with BRCA1 or BRCA2 mutations detected in case series unselected for family history: a combined analysis of 22 studies. American Journal of Human Genetics 2003;**72**(5):1117-30.
2. Evans DG, Shenton A, Woodward E, et al. Penetrance estimates for BRCA1 and BRCA2 based on genetic testing in a Clinical Cancer Genetics service setting: risks of breast/ovarian cancer quoted should reflect the cancer burden in the family. BMC Cancer 2008;**8**(1):155.
3. Ford D, Easton DF, Bishop DT, et al. Risks of cancer in BRCA1-mutation carriers. The Lancet 1994;**343**(8899):692-95.
4. Easton DF, Pooley KA, Dunning AM, et al. Genome-wide association study identifies novel breast cancer susceptibility loci. Nature 2007;**447**:1087-93.
5. Gayther SA, Warren W, Mazoyer S, et al. Germline mutations of the BRCA1 gene in breast and ovarian cancer families provide evidence for. Nature Genetics 1995;**11**(428-433).
6. Loveday C, Turnbull C, Ramsay E, et al. Germline mutations in RAD51D confer susceptibility to ovarian cancer. Nature Genetics 2011;**43**(9):879-82.
7. Meindl A, Hellebrand H, Wiek C, et al. Germline mutations in breast and ovarian cancer pedigrees establish RAD51C as a human cancer susceptibility gene. Nature Genetics 2010;**42**(5):410-14.
8. Antoniou AC, Casadei S, Heikkinen T, et al. Breast-cancer risk in families with mutations in PALB2. New England Journal of Medicine 2014;**371**(6):497-506.
9. Robson ME, Storm CD, Weitzel J, et al. American Society of Clinical Oncology policy statement update: genetic and genomic testing for cancer susceptibility. Journal of Clinical Oncology 2010;**28**(5):893-901.
10. Wacholder S, Hartge P, Prentice R, et al. Performance of common genetic variants in breast-cancer risk models. New England Journal of Medicine, 2010;**362**(11):986-93.
11. Manchanda R, Legood R, Burnell M, et al. Cost-effectiveness of population screening for BRCA mutations in Ashkenazi Jewish women compared with family history–based testing. Journal of the National Cancer Institute 2015;**107**(1):380.
12. Manchanda R, Loggenberg K, Gessler S, et al. Population based testing for high-penetrance dominant gene mutations: initial results from the pilot phase of GCaPPS. International Journal of Gynaecological Cancer 2012;**22**:S42-S43.
13. Gabai-Kapara E, Lahad A, Kaufman B, et al. Population-based screening for breast and ovarian cancer risk due to BRCA1 and BRCA2. Proceedings of the National Academy of Sciences 2014;**111**(39):14205-10.
14. King M, Levy-Lahad E, Lahad A. Population-based screening for brca1 and brca2: 2014 lasker award. Journal of the American Medical Association 2014;**312**(11):1091-92.
15. Levy-Lahad E, Lahad A, King MC. Precision Medicine Meets Public Health: Population Screening for BRCA1 and BRCA2. Journal of the National Cancer Institute 2015;**107**(1):420.
16. Annas GJ, Elias S. 23andMe and the FDA. New England Journal of Medicine 2014;**370**(11):985-88.
17. McGuire AL, Joffe S, Koenig BA, et al. Ethics and genomic incidental findings. Science 2013;**340**:1047-48.
18. Schuurman AG, van der Kolk DM, Verkerk MA, et al. Maximising the efficiency of clinical screening programmes: balancing predictive genetic testing with a right not to know. European Journal of Human Genetics 2015;**23**:1124-28.
19. Su Y, Borry P, Otte IC, et al. "It's our DNA, we deserve the right to test!" A content analysis of a petition for the right to access direct-to-consumer genetic testing. Personalized Medicine 2013;**10**(7):729-39.
20. Heshka JT, Palleschi C, Howley H, et al. A systematic review of perceived risks, psychological and behavioral impacts of genetic testing. Genetics in Medicine 2008;**10**(1):19-32.
21. Bosch N, Junyent N, Gadea N, et al. What factors may influence psychological well being at three months and one year post BRCA genetic result disclosure? The Breast 2012;**21**:755-60.
22. Ringwald J, Wochnowski C, Bosse K, et al. Psychological Distress, Anxiety, and Depression of Cancer-Affected BRCA1/2 Mutation Carriers: a Systematic Review. Journal of Genetic Counseling 2016;**25**:880-91.
23. Meiser B. Psychological impact of genetic testing for cancer susceptibility: An update of the literature. Psycho-Oncology 2005;**14**(12):1060-74.

For peer review only

24. Larouche G, Chiquette J, Pelletier S, et al. Do women change their breast cancer mammogram screening behaviour after BRCA1/2 testing? Familial Cancer 2016:1-6.
25. Beery TA, Williams JK. Risk reduction and health promotion behaviors following genetic testing for adult-onset disorders. Genetic Testing 2007;**11**(2):111-23.
26. Marteau TM, French DP, Griffin SJ, et al. Effects of communicating DNA-based disease risk estimates on risk-reducing behaviours. The Cochrane Library 2010.
27. Hollands GJ, French DP, Griffin SJ, et al. The impact of communicating genetic risks of disease on risk-reducing health behaviour: systematic review with meta-analysis. British Medical Journal 2016;**352**:i1102.
28. Biesecker BB, Ishibe N, Hadley DW, et al. Psychosocial factors predicting BRCA1/BRCA2 testing decisions in members of hereditary breast and ovarian cancer families. American Journal of Medical Genetics 2000;**93**(4):257-63.
29. Caulfield T, McGuire A, L. . Direct-to-consumer genetic testing: perceptions, problems, and policy responses. Annual Review of Medicine 2012;**63**:23-33.
30. Caulfield T, Ries NM, Ray PN, et al. Direct-to-consumer genetic testing: good, bad or benign? Clinical Genetics 2010;**77**(2):101-05.
31. Matloff E, Caplan A. Direct to confusion: lessons learned from marketing BRCA testing. The American Journal of Bioethics 2008;**8**(6):5-8.
32. George A, Riddell D, Seal S, et al. Implementing rapid, robust, costeffective, patient-centred, routine genetic testing in ovarian cancer patients. bioRxiv 2016.
33. Plaskocinska I, Shipman H, Drummond J, et al. New paradigms for BRCA1/BRCA2 testing in women with ovarian cancer: results of the Genetic Testing in Epithelial Ovarian Cancer (GTEOC) study. Journal of Medical Genetics 2016.
34. Zilliacus EM, Meiser B, Lobb EA, et al. Are videoconferenced consultations as effective as face-to-face consultations for hereditary breast and ovarian cancer genetic counseling? Genetics in Medicine 2011;**13**(11):933-41.
35. Buchanan AH, Datta SK, Skinner CS, et al. Randomized trial of telegenetics vs. in-person cancer genetic counseling: Cost, patient satisfaction and attendance. Journal of Genetic Counseling 2015;**24**(6):961-70.
36. Meropol NJ, Daly MB, Vig HS, et al. Delivery of Internet-based cancer genetic counselling services to patients' homes: a feasibility study. Journal of Telemedicine and Telecare 2011;**17**:36–40.
37. Lerman C, Croyle RT, Tercyak KP, et al. Genetic testing: psychological aspects and implications. Journal of Consulting and Clinical Psychology 2002;**70**(3):784-97.
38. Ropka ME, Wenzel J, Phillips EK, et al. Uptake rates for breast cancer genetic testing: a systematic review. Cancer Epidemiology Biomarkers & Prevention 2006;**15**(5): 840-55.
39. Meisel SF, Side L, Fraser L, et al. Population-based, risk-stratified genetic testing for ovarian cancer risk: a focus group study. Public Health Genomics 2013;**16**(4):184-91.
40. Henneman L, Timmermans DR, Bouwman CM, et al. 'A low risk is still a risk': exploring women's attitudes towards genetic testing for breast cancer susceptibility in order to target disease prevention. Public Health Genomics 2011;**14**(4-5):238-47.
41. Ulrich CM, Kristal AR, White E, et al. Genetic testing for cancer risk: A population survey on attitudes and intention. Public Health Genomics 1998;**1**(14):213-22.
42. Henneman L, Vermeulen E, van El CG, et al. Public attitudes towards genetic testing revisited: comparing opinions between 2002 and 2010. European Journal of Human Genetics 2013;**21**(8):793-99.
43. Meisel SF, Pashayan N, Rahman B, et al. Adjusting the frequency of mammography screening on the basis of genetic risk: Attitudes among women in the UK. The Breast 2015;**24**(3):237-41.
44. Durfy SJ, Page A, Eng B, et al. Attitudes of high school students toward carrier screening and prenatal diagnosis of cystic fibrosis. Journal of Genetic Counseling 1994;**3**(2):141-55.
45. Etchegary H, Cappelli M, Potter B, et al. Attitude and knowledge about genetics and genetic testing. Public Health Genomics 2010;**13**(2):80-88.
46. Vermeulen E, Henneman L, van El CG, et al. Public attitudes towards preventive genomics and personal interest in genetic testing to prevent disease: a survey study. The European Journal of Public Health 2013;**24**:768-75.

For peer review only

47. Howard HC, Knoppers BM, Cornel MC, et al. Whole-genome sequencing in newborn screening: A statement on the continued importance of targeted approaches in newborn screening programmes. European Journal of Human Genetics 2015;**23**:1593-600.
48. Knoppers BM, Sénécal K, Borry P, et al. Whole-genome sequencing in newborn screening programs. Science Translational Medicine 2014;**6**:229cm2.
49. Waisbren SE, Bäck DK, Liu C, et al. Parents are interested in newborn genomic testing during the early postpartum period. Genetics in Medicine, 2015;**17**:501–04.
50. Singer E. Public attitudes toward genetic testing. Population Research and Policy Review 1991;**10**(3):235-55.
51. Henneman L, Timmermans DRM, Wal GVD. Public Attitudes Toward Genetic Testing: Perceived Benefits and Objections. Genetic Testing 2006;**10**(2):139-45.
52. Gottfredson LS. Intelligence: is it the epidemiologists' elusive" fundamental cause" of social class inequalities in health? Journal of Personality and Social Psychology 2004;**86**(1).
53. Beeken RJ, Simon AE, von Wagner C, et al. Cancer fatalism: deterring early presentation and increasing social inequalities? Cancer Epidemiology Biomarkers & Prevention 2011;**20**(10):2127-31.
54. von Wagner C, Good A, Whitaker KL, et al. Psychosocial determinants of socioeconomic inequalities in cancer screening participation: a conceptual framework. Epidemiologic Reviews 2011;**33**(1):135-47.
55. Sanderson SC, Waller J, Jarvis MJ, et al. Awareness of lifestyle risk factors for cancer and heart disease among adults in the UK. Patient Education and Counseling 2009;**74**(2):221-27.
56. Lo SH, Waller J, Wardle J, et al. Comparing barriers to colorectal cancer screening with barriers to breast and cervical screening: a population-based survey of screening-age women in Great Britain. Journal of Medical Screening 2013;**20**(2):73-79.
57. Dent T, Jbilou J, Rafi I, et al. Stratified cancer screening: the practicalities of implementation. Public Health Genomics 2013;**16**(3):94-99.
58. Hall AE, Chowdhury S, Hallowell N, et al. Implementing risk-stratified screening for common cancers: a review of potential ethical, legal and social issues. Journal of Public Health 2014;**36**(2):285-91.
59. Lerman C, Daly M, Masny A, et al. Attitudes about genetic testing for breast-ovarian cancer susceptibility. Journal of Clinical Oncology 1994;**12**(4):843-50.
60. Bottorff JL, Ratner PA, Balneaves LG, et al. Women’s interest in genetic testing for breast cancer risk the influence of sociodemographics and knowledge. Cancer Epidemiology Biomarkers & Prevention 2002;**11**(1):89-95.
61. Aro AR, Hakonen A, Hietala M, et al. Acceptance of genetic testing in a general population: age, education and gender differences. Patient Education and Counseling 1997;**32**(1):41-49.
62. Borzekowski DL, Guan Y, Smith KC, et al. The Angelina effect: immediate reach, grasp, and impact of going public. Genetics in Medicine 2013;**16**(7):516-21.
63. Kamenova K, Reshef A, Caulfield T. Angelina Jolie's faulty gene: newspaper coverage of a celebrity's preventive bilateral mastectomy in Canada, the United States, and the United Kingdom. Genetics in Medicine 2013;**16**:522-28.
64. Cameron LD, Reeve J. Risk perceptions, worry, and attitudes about genetic testing for breast cancer susceptibility. Psychology & Health 2006;**21**(2):211-30.
65. Lipkus IM, Klein WM, Skinner CS, et al. Breast cancer risk perceptions and breast cancer worry: What predicts what? Journal of Risk Research 2005;**8**(5):439-52.
66. Sheeran P, Webb TL. The Intention–Behavior Gap. Social and Personality Psychology Compass 2016;**10**(9):503-18.
67. Gilovich T, Griffin D, Kahneman D. *Heuristics and biases: The psychology of intuitive judgment*: Cambridge University Press, 2002.
68. Wilson TD, Gilbert DT. Affective forecasting. Advances in Experimental Social Psychology 2003;**35**:345-411.
69. Sanderson SC, Wardle J. Associations between anticipated reactions to genetic test results and interest in genetic testing: Will Self-Selection Reduce the Potential for Harm? Genetic Testing 2008;**12**(1):59-66.

For peer review only

Table 1: Sample demographic and personal characteristics (n=837)

	N (%)
**Gender**	
Female	837 (100.0)
**Age (mean, SD)**	45.9 (15.5)
**Age group**	
18-29 years	143 (17.1)
30-39 years	179 (21.4)
40-49 years	160 (19.1)
50-64	234 (28.0)
65-74 years	121 (14.5
**Ethnicity**	
White British	725 (86.6)
White Other	52 (6.3)
Mixed ethnicity	12 (1.4)
Black	11 (1.3)
South Asian	28 (3.3)
Other	9 (1.1)
**Education**	
No formal qualifications	126 (15.1)
Less than degree level qualification	478 (57.1)
Degree level (or equivalent)	233 (27.8)
**Marital status**	
Married/ Civil partnership/ Cohabiting	459 (54.8)
Single	177 (21.1)
Divorced/ Separated	140 (16.7)
Widowed	61 (7.3)
**Family history of cancer (mother, father, brother, sister)**	
Yes	305 (36.4)
No/don't know	532 (63.6)
**Perceived chances of getting ovarian cancer compared to other women of same age**	
Much higher/higher	64 (7.6)
About the same as others	590 (70.5)
Much lower/lower	183 (21.9)
**Perceived chances of getting breast cancer compared to other women of same age**	
Much higher/higher	83 (9.9)
About the same as others	616 (73.7)
Much lower/lower	137 (16.4)

For peer review only

Table 2. Univariate Analyses of anticipated reactions to risk disclosure of breast and ovarian cancer genetic risk

Variable	'I would try harder to have a healthy lifestyle'[1]		'It would give me more control over my life'[1]		'I would feel that it wasn't worth making lifestyle changes'[1]		'I would feel less free to make choices in my life'[1]	
	n (%) agree/ strongly agree	p-value	n (%) agree/ strongly agree	p-value	n (%) agree/ strongly agree	p-value	n (%) agree/ strongly agree	p-value
**Sample total (n = 925)**	669 (71.9)		506 (54.7)		130 (14.1)		152 (16.4)	
**Age**								
**>50**	289 (70.0)	0.235	237 (57.8)	0.091	60 (14.6)	0.670	62 (15.1)	0.337
**≤50**	380 (73.5)		269 (52.2)		70 (13.6)		90 (17.1)	
**Ethnicity**								
**White**	624 (71.8)	0.741	468 (54.2)	0.218	122 (14.1)	0.827	**136 (15.7)**	**0.033***
**Ethnic minority**	45 (73.8)		38 (62.3)		8 (13.1)		**16 (26.2)**	
**Education**								
**University degree**	172 (68.8)	0.197	**123 (49.2)**	**0.041***	**20 (8.0)**	**0.001***	36 (14.4)	0.310
**Below university degree**	497 (73.1)		**383 (56.7)**		**110 (16.3)**		116 (17.2)	
**Marital status**								
**Married/cohabiting**	372 (74.4)	0.071	**291 (58.4)**	**0.014***	65 (13.1)	0.358	72 (14.4)	0.075
**Single/widowed/divorced**	297 (69.1)		**215 (50.4)**		65 (15.2)		80 (18.8)	
**Family history of any cancer**								
**No/don't know**	260 (72.4)	0.781	**286 (51.1)**	**0.004***	51 (14.3)	0.798	98 (17.5)	0.198
**Yes**	403 (71.6)		**217 (60.8)**		77 (13.7)		51 (14.3)	
**Perceived risk of OC (n = 847)**								
**Much lower than others**	42 (66.7)	0.056	31 (50.0)	0.324	12 (19.0)	0.696	**23 (37.1)**	**<0.001***
**Lower than others**	97 (77.0)		66 (53.7)		18 (14.4)		**25 (20.2)**	
**The same as others**	430 (72.3)		318 (53.4)		82 (13.8)		**87 (14.6)**	
**Higher than others**	48 (81.4)		39 (66.1)		7 (11.9)		**10 (16.9)**	
**Much higher than others**	1 (25.0)		3 (75.0)		0 (0.0)		**1 (25.0)**	
**Perceived risk of BC (n = 847)**								
**Much lower than others**	17 (65.4)	0.367	9 (34.6)	0.350	7 (26.9)	0.322	**9 (34.6)**	**0.023***
**Lower than others**	90 (79.6)		60 (54.1)		14 (12.5)		**27 (24.3)**	
**The same as others**	448 (71.7)		342 (54.9)		84 (13.5)		**95 (15.2)**	
**Higher than others**	57 (76.0)		43 (57.3)		13 (17.3)		**14 (18.7)**	
**Much higher than others**	7 (77.8)		5 (55.6)		1 (11.1)		**2 (22.2)**	

[1]Each statement preceded by: If I knew my risk of breast and ovarian cancer, I would…; Abbreviations: BC = breast cancer, OC = ovarian cancer

* Significant at p <0.05

For peer review only

Table 3. Multivariate logistic regression of demographic and personal predictors of anticipated reactions to disclosure of genetic risk of breast and ovarian cancer

Variable	'I would try harder to have a healthy lifestyle'[1]			It would give me more control over my life'[1]			'I would feel it wasn't worth making lifestyle changes'[1]			'I would feel less free to make choices in my life'[1]		
	OR	95% CI	p-value	OR	95% CI	p-value	OR	95% CI	p-value	OR	95% CI	p-value
**Age**												
**>50**	1			1			1			1		
**≤50**	1.86	0.84-1.66	0.321	0.80	0.60-1.11	0.205	0.99	0.64-1.53	0.988	1.22	0.81-1.85	0.333
**Ethnicity**												
**White**	1			1			1			1		
**Ethnic minority**	0.95	0.51-1.66	0.859	1.51	0.86-2.64	0.149	1.01	0.45-2.22	0.985	1.60	0.86-3.01	0.138
**Education**												
**University degree**	1			1			**1**			1		
**Below university degree**	1.35	0.96-1.91	0.081	1.39	1.01-1.91	0.039	**1.99**	**1.19-3.35**	**0.009***	1.32	0.85-2.03	0.333
**Marital status**												
**Single/widowed/divorced**	1			1			1			1		
**Married/cohabiting**	1.34	0.98-1.83	0.062	1.41	1.07-1.87	0.016	0.92	0.62-1.38	0.704	0.69	0.50-1.01	0.051
**Family history of any cancer**												
**No/don't know**	1			1			1			1		
**Yes**	1.14	0.81-1.61	0.438	1.31	0.96-1.78	0.085	1.09	0.70-1.68	0.695	0.81	0.54-1.24	0.345
**Perceived risk of OC**												
**Much lower/lower than others**	1			1			1			1		
**The same as others**	1.15	0.68-1.95	0.588	0.95	0.58-1.55	0.848	0.90	0.47-1.72	0.759	0.55	0.30-0.99	0.046
**Higher/much higher than others**	1.41	0.64-3.15	0.392	1.86	0.90-3.84	0.092	0.54	0.19-1.51	0.241	0.61	0.25-1.48	0.276
**Perceived risk of BC**												
**Much lower/lower than others**	1			1			1			1		
**The same as others**	0.65	0.36-1.19	0.171	1.29	0.75-2.22	0.349	0.95	0.46-1.99	0.899	0.81	0.43-1.55	0.538
**Higher/much higher than others**	0.70	0.32-1.54	0.376	0.97	0.49-1.94	0.934	1.44	0.57-3.63	0.431	0.99	0.43-2.33	0.996

[1]Each statement was preceded by: If I knew my risk of breast and ovarian cancer…

Abbreviations: BC = breast cancer, OC = ovarian cancer

***** Significant after Bonferroni correction at p <0.012

For peer review only

BMJ Open

Anticipated health behaviour changes and perceived control in response to disclosure of genetic risk of breast and ovarian cancer: a quantitative survey study among women in the UK

Journal:	*BMJ Open*
Manuscript ID	bmjopen-2017-017675.R1
Article Type:	Research
Date Submitted by the Author:	13-Sep-2017
Complete List of Authors:	Meisel, Susanne; Kings College London<br>Fraser, Lindsay; Institute for Women's Health, Department of Women's Cancer<br>Side, Lucy; University College London Institute for Women's Health<br>Gessler, Sue; University College London Hospitals NHS Foundation Trust, ; UCL Institute for Women’s Health and NIHR University College London Hospitals Biomedical Research Centre, Department of Women’s Cancer<br>Hann, Katie; University College London Institute for Women's Health, Department of Women's Cancer<br>Wardle, Jane; University College London, Dept. of Epidemiol<br>Lanceley, Anne; UCL, Department of Women's Cancer
**Primary Subject Heading**:	Genetics and genomics
Secondary Subject Heading:	Public health
Keywords:	Breast cancer, Ovarian cancer, Predictive genetic testing, Risk

SCHOLARONE™ Manuscripts

For peer review only

TITLE: Anticipated health behaviour changes and perceived control in response to disclosure of genetic risk of breast and ovarian cancer: a quantitative survey study among women in the UK

Authors: Susanne F Meisel PhD[1], Lindsay Fraser BSc[2], Lucy Side MD[2], Sue Gessler PhD[2], Katie E J Hann MSc[2], Jane Wardle PhD[3]† and Anne Lanceley PhD[2] for the PROMISE study team

[1]Institute of Psychiatry, Psychology and Neuroscience, King's College London, London, UK.

[2]Department of Women's Cancer, EGA UCL Institute for Women's Health, University College London, London, UK.

[3]Department of Behavioural Science and Health, Institute of Epidemiology and Health Care, University College London, London, UK.

Corresponding Author:

Anne Lanceley, PhD

237c

Medical School Building

74 Huntley Street

London, WC1E 6AU

Email: a.lanceley@ucl.ac.uk

Phone: +4420 7679 6807

For peer review only

Abstract

Background: Genetic risk assessment for breast cancer and ovarian cancer (BCOC) is expected to make major inroads into mainstream clinical practice. It is important to evaluate the potential impact on women ahead of its implementation in order to maximise health benefits, as predictive genetic testing without adequate support could lead to adverse psychological and behavioural responses to risk disclosure.

Objective: To examine anticipated health behaviour changes and perceived control to disclosure of genetic risk for BCOC, and establish demographic and person-specific correlates of adverse anticipated responses in a population-based sample of women.

Design: Cross-sectional quantitative survey study carried out by the UK Office for National Statistics in January and March 2014.

Setting: Face-to-face computer-assisted interviews conducted by trained researchers in participants' homes.

Participants: 837 women randomly chosen from households across the UK identified from the Royal Mail's Postcode Address File.

Outcome measures: Anticipated health behaviour change and perceived control to disclosure of BCOC risk.

Results: In response to a genetic test result, most women (72%) indicated 'I would try harder to have a healthy lifestyle' and over half (55%) felt 'it would give me more control over my life'. These associations were independent of demographic factors or perceived risk of BCOC in Bonferroni-corrected multivariate analyses. However, a minority of women (14%) felt 'it isn't worth making lifestyle changes', and that 'I would feel less free to make choices in my life' (16%) in response to BCOC risk disclosure. The former belief was more likely to be held by women who were educated below university degree level (p <0.001) after adjusting for other demographic and person-specific correlates.

For peer review only

Conclusion: These findings indicate that women in the UK largely anticipate that they would engage in positive health behaviour changes in response to BCOC risk disclosure.

Strengths and limitations of this study

- Stratified random sampling, the 'gold-standard' for survey recruitment, was used in this study.
- The study reports on anticipated responses to genetic BCOC risk information from a large general population sample of women living in the UK.
- The survey questions were broad, and may have been interpreted differently by different participants
- A general hypothetical scenario about receiving genetic BCOC risk information was used instead of asking participants to consider a hypothetical scenario of receiving a high or low risk genetic test result.

For peer review only

Source of funding:

This research was funded by grants from The Eve Appeal (London, UK, grant code: 509050) and Cancer Research UK (London, UK; grant code: 508007). Jane Wardle was supported by Cancer Research UK as part of the CR UK-UCL Cancer Centre.

Conflicts of interest:

All authors have completed the ICMJE uniform disclosure form at www.icmje.org/coi_disclosure.pdf and declare: SFM and KEJH had financial support from Cancer Research UK and the Eve Appeal for the submitted work; no financial relationships with any organisations that might have an interest in the submitted work in the previous three years; no other relationships or activities that could appear to have influenced the submitted work.

Authors contributions:

SFM participated in study design, analysed the data and drafted the manuscript; KEJH helped with data interpretation and critically revised the manuscript; LS, LF, SG, AL and JW conceived the study, participated in study design and coordination and critically revised the manuscript. All authors have seen and approved the final manuscript except JW who sadly died before submission of the manuscript.

Data sharing statement

The data is available on request from the corresponding author: a.lanceley@ucl.ac.uk

Word count: 4014

Number of tables: 3 main and 1 supplementary

For peer review only

Introduction

Advances in genetic technologies have led to the identification of gene mutations associated with an increased risk of developing breast cancer and ovarian cancer (BCOC). In addition to germline mutations in *BRCA 1* and *BRCA 2*, which confer a very high lifetime risk of BCOC (39%- 65% and 11%-37%, respectively) [1-3], a growing list of moderate- and lower-risk markers specific to each cancer type have been identified [4-10]. These influence cancer development in a larger proportion of the population despite small effect sizes. It is anticipated that, in future, some moderate risk markers will be included in existing panel tests used in clinical genetics practice[11-13]. While high penetrance genes like *BRCA1* and *BRCA2* confer a lifetime risk for cancer of 50% or greater, the lifetime risk for moderately penetrant genes ranges from 20% to 50% and low penetrance genes are thought to have a limited effect[14].

To date, genetic testing of *BRCA1* and *BRCA2* is conducted in clinical practice only, in individuals with a strong family history, or after a cancer diagnosis. However, it has been argued that this approach is likely to miss a substantial proportion of women at high risk (~30%), particularly if the family is small, the mutation was transmitted through the paternal line, or the individual has a number of lower risk mutations whose effects are additive[15-18]. Given that genetic testing for BCOC risk can now be carried out at a relatively low cost, there have been calls to make it available on a population level, based on the premise that timely awareness of genetic risk would empower individuals to make informed decisions about cancer risk management, allow for early intervention, and ultimately, improve clinical outcomes[19]. There is a drive from healthcare professionals to 'mainstream' genetic testing as part of routine cancer care in oncology services, aimed at improving diagnosis and providing individualised treatment for patients (http://www.mcgprogramme.com/)[20-23]. *BRCA1* and *BRCA2* testing may be offered to women diagnosed with triple negative breast cancer and information on genetic status is likely to influence clinical care and management of the patient and the family[24-25]. Moreover, genetic testing for BCOC susceptibility can already be purchased over the Internet[26] (www.23andme.com; www.getcolor.com). The notion that individuals have a 'right to know' their genetic information has also gained traction in recent years[27-30].

For peer review only

However, concerns persist about patient safety related to poor psychological adjustment following genetic risk disclosure. Studies investigating reactions to disclosure of BRCA status commonly do not find evidence for persistent negative psychological outcomes beyond one year after result disclosure[31-34]. There is also some evidence suggesting that genetic test results indicating a higher risk of disease can lead to behaviour change such as uptake of breast and colorectal cancer screening[31 35 36], although two systematic reviews found no significant impact of genetic risk information on other behaviours[37 38] including smoking, physical activity, use of medication or vitamins, diet, alcohol use and use of a sunscreen. However, genetic testing has hitherto only been performed in women with a strong family history of BCOC, so these results may not generalise to a population largely unaware of any increased risk.

Currently, one-to-one genetic counselling is strongly embedded within clinical genetic testing services, offering education and support throughout the process. If genetic testing were introduced on a population level, the current model of delivering genetic counselling would no longer be feasible. It has been argued that returning genetic risk test results for highly debilitating conditions such as BCOC without appropriate professional guidance would leave room for misinterpretation and uncertainty, increasing the risk of adverse psychological outcomes[39-42]. In recent years, research has begun to investigate new approaches of providing cancer genetic services. Mainstreaming genetic testing, by offering women with a diagnosis of ovarian cancer testing directly through the oncology clinic and providing post-test genetic counselling via clinical genetics for those with a hereditary gene mutation, is one example[43-44]. Research has found that mainstreaming genetic testing in this way can successfully reduce time and resources spent on the processes[45] and does not appear to negatively affect patients' psychological well-being[46]. Genetic counselling with patients and healthy family members via telephone

For peer review only

or video consultations instead of face-to-face genetic counselling has also been found to be feasible and patients are generally satisfied[47-49]. These new approaches to offering genetic testing and providing genetic counselling for cancer susceptibility may be advantageous in future if testing is offered to patients more widely.

Although a wealth of studies have investigated anticipated psychological reactions to disclosure of BCOC genetic risk in higher risk groups[31 50 51] little is known about this issue in women unselected for family history. One qualitative study found that women in the UK would support the idea of genetic testing for risk of ovarian cancer because they expected it to trigger positive lifestyle changes and provide a means of control over cancer development[52]. However, in a similar study investigating attitudes towards genetic testing for breast cancer risk in the Netherlands, women were more ambivalent about disclosure of genetic risk; although there was recognition of the benefits of testing, some women were uncertain about their reactions to risk disclosure and the impact it would have on their lives[53].

An early population-based survey investigated attitudes towards genetic testing for breast cancer among 836 women living in Washington State[54]. Results showed that over three quarters of the sample expected that awareness of their genetic risk of breast cancer would give them more control over their life, and a similar number reported that they would 'definitely' or 'probably' take part in testing if it was offered to them. A more recent population-based study with over 800 participants in the Netherlands compared attitudes towards genetic testing between 2002 and 2010[55]. Results showed that positive expectations about the utility of genetic testing had increased over the 8-year interval. However, about a third of participants believed that awareness of genetic risk would deprive people of the freedom to live as they want, and this proportion remained unchanged over time. Although these findings give an indication about the acceptability of population-based genetic testing, they may be culture-specific and

For peer review only

may therefore not generalise to other populations. Furthermore, the latter survey asked only about attitudes towards genetic testing more broadly; attitudes to genetic risk disclosure specific to BCOC may be different.

The aim of the present study was to examine the anticipated health behaviour changes and perceived control in response to disclosure of genetic risk for BCOC and to explore demographic and person-specific correlates (for example marital status and family history of cancer) of anticipated response among a population sample of women in the UK. Identifying subgroups who are more likely to anticipate a negative response to learning about their genetic risk of developing these cancers, before genetic testing is integrated into mainstream healthcare (e.g. by including it in current cancer screening programmes), gives scope for timely intervention to avoid further increasing disparities in healthcare.

Methods and Procedure

This quantitative study complied with the principles set out in the declaration of Helsinki. As a population-based anonymous survey, it was exempt from ethical approval, in accordance with the guidelines set out by the UCL Ethics committee for non-NHS research (http://ethics.grad.ucl.ac.uk/exemptions.php).

Sample

Data were collected by the UK Office for National Statistics (ONS) in their monthly survey which is conducted on behalf of a range of organisations (e.g. government departments, non-governmental institutions and academic departments). Each month, 2010 households are identified from the Royal Mail's Postcode Address File using stratified random probability sampling. Selected addresses are contacted up to eight times at different times and days of the week to maximize response rates. One

For peer review only - http://bmjopen.bmj.com/site/about/guidelines.xhtml

person aged over 16 from each household is randomly chosen to complete a computer-assisted face-to-face interview with trained researchers. Surveys commonly include a variety of different topics, making participation bias unlikely, although participants can opt out at any point. Questions on the 'genetics and screening' module were included in data collection waves in January and March 2014.

Measures

The 'genes and cancer' module was introduced with a short statement: '*Genes contain the 'instruction manual' of life, called DNA. Genes are passed from parents to their children. Nowadays, it is possible to predict whether someone is likely to develop certain diseases by looking at their genes. This is called genetic testing'*. No further information was given, as we were interested in women's attitudes towards BCOC genetic testing based on their current understanding of it. Participants were asked several questions in relation to genetic testing for BCOC risk and attitudes on breast cancer screening (see supplementary material for detail). Some of the findings have previously been published[56].

Outcome variables

Anticipated reactions to learning about increased BCOC risk were assessed with statements ranging from positively framed outcomes such as the opportunity to exert personal control ('If I knew my genetic risk of breast cancer and ovarian cancer, it would give me more control over my life') or to pursue health behaviours aimed at mitigating risk ('If I knew my genetic risk of breast cancer and ovarian cancer I would try harder to have a healthy lifestyle'), to more negatively framed outcomes such as curtailing freedom of choice ('If I knew my genetic risk of breast cancer and ovarian cancer, I would feel less free to make choices in my life') or the perceived futility of attempting behaviour change ('If I knew my genetic risk of breast cancer and ovarian cancer, I would feel it wasn't worth making

For peer review only

lifestyle changes'). All items were scored on a five-point Likert scale ranging from 'strongly disagree'-'strongly agree', and were adapted from previous research[54 55].

Predictor variables

Perceived risk of BCOC was each assessed with one question 'Compared with other women of your age, what do you think are your chances of getting breast [ovarian] cancer' with response options of: 'much lower than others', 'lower than others', 'the same as others', 'higher than others', 'much higher than others'. Given that individual risk categories pertaining to much lower/much higher perceived risk were small, perceived risk was grouped into three categories: 'much lower/lower than others; 'same as others', and 'higher/much higher than others'.

Age, ethnicity, educational attainment and marital status were derived using standard survey items (https://tinyurl.com/ya6gn2g6). Age was coded as ≤50 vs. >50 years to investigate any generational differences in anticipated reactions and attitudes. We chose 50 years of age as the cut-off point since public interest in the UK for genetic testing has been reported as being greatest for people aged 46-60 years[57] and we felt that the age of 50, being close to the average age of menopause in the UK (which is 51), was a reasonable mid-point to assess generational effects. Ethnicity was classified as 'White' vs. 'ethnic minority' because the individual ethnic minority sub-groups were small. Educational attainment was classified as university degree or equivalent vs. below university degree level. Marital status was coded as married/cohabiting vs. single/widowed/divorced. Family history of any cancer was assessed with one question: 'Have your mother, father, or any of your brothers or sisters, been diagnosed with cancer?' with response options being 'yes', 'no', and 'don't know'.

Statistical analyses

For peer review only

We included women aged 18-74 to reflect the population to whom genetic testing for risk of BCOC would likely become available in the future. Statistical analyses were carried out using the Statistical Package for Social Sciences (SPSS) version 20.0 (SPSS Inc., Chicago, IL). Descriptive statistics were explored using frequency tables. We explored demographic (age, ethnicity, education) and person-specific predictors (marital status, family history of cancer, perceived risk of ovarian cancer, perceived risk of breast cancer) for each outcome variable using χ^2 tests. Multivariate logistic regression models were used to investigate the demographic and person-specific correlates of anticipated reactions to disclosure of personal BCOC risk. Bonferroni corrections were employed in all multivariate analyses to correct for multiple testing, with $\alpha = 0.0125$.

Results

Demographic and personal characteristics

Data for the current study were collected in two waves: January and March 2014. In the January wave, 8% (n = 166) of the 2,010 selected households were not eligible because they were businesses or empty properties. Of 1,844 eligible households, 9% (n = 171) could not be contacted and 33% (n = 608) declined to take part in the ONS survey. In the March wave, 1,853 households were eligible (92%). Of those, 13% (n = 237) could not be contacted and 31% (n = 578) chose not to take part. Therefore, the overall response rate was 57%, comparable to previous ONS surveys (ONS 2013; ONS 2012; http://www.ons.gov.uk/ons/index.html). The total female sample was n=1,095.

After excluding participants with incomplete data for any of the outcome variables (n= 120), or who were aged younger than 18 or older than 74 years (n = 138), the final sample for analysis consisted of 837 women. Table 1 presents the demographic and personal characteristics of the sample. In common

For peer review only

with other survey studies, education was higher in the sample than in the general population; other demographic characteristics were comparable to the UK population of women aged 18-74 (ONS Census 2011: (http://www.ons.gov.uk/ons/guide-method/census/2011/index).

Although 36.4% (n = 305) of the sample reported a family history of any cancer, most women felt that they were not particularly at risk of breast or ovarian cancer; only 9.9% (n = 83) thought they were at ‘higher’ or ‘much higher’ than average risk of breast cancer, and even fewer (7.6%, n = 64) thought they were at ‘higher’ or ‘much higher’ risk of ovarian cancer. A full breakdown of responses can be found in the supplementary material Table 1.

Support of genetic testing

Overall, women were supportive of the idea of genetic testing for breast and ovarian cancer. Only 2.0% (n = 18) thought that it was ‘never’ a good time to have a genetic test. However, about a third of the sample (31.4%, n = 290) were ‘unsure’. Opinions were divided on when the best time would be to get tested, with 8.3% (n = 77) thinking that it would be ‘just after birth’, just over a quarter (27.4%, n = 253) thought ‘during school’, 10.6% (n = 98) reported ‘just before marriage’, 16.9% (n=156) ‘before they plan to have a child’, and 3.4% (n = 31) thought the best time would be ‘when they are unwell’. There were no demographic or personal correlates with ‘ever’ vs. ‘never’ supporting genetic testing for breast and ovarian cancer (data not shown).

Anticipated response to disclosure of genetic risk of breast and ovarian cancer

Overall, women anticipated to respond proactively to awareness of their genetic risk. Most (71.9%, n = 669) said that they ‘would try harder to have a healthy lifestyle’, and over half (54.7%, n = 506) anticipated that it would give them ‘more control over my life’. However, a minority reported that it

For peer review only

would 'not be worth making lifestyle changes' (14.1%, n = 130) in response to genetic risk disclosure, and that they would feel 'less free to make choices in my life' (16.4%, n = 152).

In univariate analyses, there were no differences across demographic and person-specific variables in the expectation that disclosure of genetic risk of BCOC would lead to increased personal efforts 'to have a healthy lifestyle' (Table 2). Women who had fewer years of formal education (χ^2 (1) = 4.57, p = 0.032), were married (χ^2 (1) = 4.53, p = 0.033), or had a family history of cancer (χ^2 (1) = 5.51, p = 0.019) were significantly more likely to report that awareness of genetic risk would give them more control over their life (Table 2). However, none of these associations remained after adjustment for covariates and multiple testing (Table 3).

Women with fewer years of formal education were also significantly more likely to agree that 'it wouldn't be worth making lifestyle changes' in response to genetic risk disclosure (χ^2 (1) = 7.17, p = 0.007), and this association remained in multivariate analyses (OR = 1.99, 95% CI = 1.19-3.35, p = 0.009). Women from ethnic minority backgrounds (χ^2 (1) = 4.18, p = 0.041), those who perceived themselves at a (much) lower or (much) higher risk of breast cancer (χ^2 (4) = 11.08, p = 0.026) or ovarian cancer (χ^2 (4) = 22.38, p<0.001) were more likely to report that awareness of genetic risk would make them 'feel less free to make choices in their life', but none of these findings was maintained after adjusting for demographic and person-specific factors, and for multiple testing (Table 3).

Discussion

This study investigated demographic and person-specific correlates of anticipated health behaviour changes and perceived control in response to disclosure of genetic risk of BCOC in a population-based sample of women in the UK.

For peer review only

Although our results suggested that most women did not expect to be negatively affected by knowing their genetic risk status for BCOC, women who had fewer years of formal education were nearly twice more likely to believe that 'it would not be worth making lifestyle changes' than those who had stayed in formal education for longer. This finding supports some earlier studies into genetic determinism (the idea that genetic make-up is more important than environmental impact for expression of diseases)[58]; although not all studies have found this link[54 59].

However, the finding that women with fewer years of formal education are less likely to believe that lifestyle changes could be effective for cancer prevention also echoes studies that investigated attitudes towards cancer screening. These studies have found that women with lower socio-economic status, which is linked to educational attainment and information literacy[60], were more likely to hold fatalistic attitudes towards cancer development. Fatalistic attitudes have in turn been implicated in lower levels of engagement with cancer screening[61-62]. It is possible that women who do not believe in the curability of cancer *per se* are also more likely to report that 'it wouldn't be worth making lifestyle changes' in response to disclosure of BCOC genetic risk, but because we did not assess beliefs about curability of BCOC, this hypothesis remains speculative. Furthermore, research has shown that those with fewer years in education are less aware of cancer risk factors such as poor diet and physical inactivity[63]. A lack of awareness that lifestyle affects a person's risk of cancer would also help to explain why some participants may feel that 'it wouldn't be worth making lifestyle changes'. Although formal years of education do not equate with health literacy, which was not measured in this study, educational attainment is strongly associated with health literacy[64], and can therefore serve as an indicator for health literacy. Given that there are already disparities in engagement with available means of early detection for cancer (e.g. screening) across the population, with lower socioeconomic status groups less

For peer review only

likely to engage[65], it will be vital to provide adequate education and information about the nature of genetic testing before it is introduced into mainstream healthcare. This point has also been highlighted by other commentators in the field[66-67]. In addition, it is important that the public has a multifaceted view of cancer and are aware of genetic and non-genetic risk factors and ways to reduce risk despite genetic susceptibility.

Although previous studies in both higher-risk[68-69] and population samples[52 70] have shown that younger women are generally more positive towards genetic testing, we found no age differences in anticipated reactions towards BCOC genetic risk disclosure. It is interesting to speculate about the impact on our study respondents about the benefits of genetic testing as a result of the spike in media coverage that took place shortly before the survey responses were collected at the beginning of 2014. The well-known actress Angelina Jolie publicly announced her reasons for undergoing BRCA genetic testing and talked about how the knowledge that she carried a gene mutation had enabled her to choose risk-reducing surgery (double mastectomy) to manage her increased risk of developing breast cancer. This celebrity revelation may have raised awareness of the potential benefits of breast (and ovarian) cancer genetic testing across all age groups[71-72], which may at least partially account for the finding that older women were as positive about genetic testing as younger women.

We found no association between participants' anticipated response to genetic risk disclosure and perceived risk of either breast or ovarian cancer. However, since few women considered themselves to be at 'higher' or 'much higher' than average risk of BCOC, sample sizes may have been too small to detect associations. Alternatively, these findings may suggest that UK women may have a more realistic understanding of their personal risk of BCOC compared to reports in previous studies from other

For peer review only

countries[73-74]. Given that all survey items were closed questions, we could not investigate this finding in more depth, but this could be done in future research.

The strengths of this study are the approach to sampling (the sample was selected using stratified random sampling which is the gold-standard of survey recruitment and therefore, self-selection is unlikely to have biased current findings) and the statistical adjustment for multiple testing, which gives confidence in the robustness of the findings. However, the study also had important limitations. We only assessed anticipated responses to disclosure of genetic risk of BCOC in general, not whether these differed in response to a hypothetical 'higher' or 'lower' genetic risk. Although all questions have been used in previous research with different samples, with comparable findings, anticipated lifestyle changes and their perceived effectiveness, could vary depending on the levels of risk conferred. Further research could explore whether attitudes to genetic risk disclosure of BCOC differ when considering different levels of risk. Secondly, because of survey constraints, we focused our investigation on a limited set of broad questions which could have been interpreted differently by participants. Questions therefore remain about exactly how respondents anticipated changing their behaviour. Future research could investigate this topic with more in-depth questions.

Naturally, responses to 'real' disclosure of genetic risk information may be less positive than our results suggest; while most participants in this study indicated anticipated making an effort to 'have a healthy lifestyle', research has shown that there is generally a 'gap' between intentions and actual behaviour[75]. In addition, given that many individuals have difficulty in accurately anticipating emotional reactions to life events[76-77], these findings can only give a broad indication about potential effects of disclosure of BCOC genetic risk. Since none of the questions were open ended, we could not explore the origins of responses in more detail, but this could be done in future research. Finally, although ethnic distribution

For peer review only - http://bmjopen.bmj.com/site/about/guidelines.xhtml

of the sample was in line with the overall population in the UK, because ethnic minority subgroups were small we were unable to conduct more detailed analyses to investigate whether attitudes to genetic testing differed among women of different ethnic minority backgrounds. It will be important to explore this in future research, particularly since a recent review found a significant lack of research investigating UK black and minority ethnic (BAME) groups awareness and attitudes toward genetic testing for cancer risk[78]. Reports that have involved UK BAME groups highlight low awareness of the availability of genetic services and fear and stigma in relation to cancer genetics[79-80].

Overall, findings from this study indicate that women in the UK are generally positive about the prospect of population based genetic testing for risk of BCOC. However, those with fewer years in education may be less positive. Given that there are already existing disparities in uptake of available means of early detection for breast cancer, careful planning is needed to avoid increasing these further. There are concerns in professional circles that predictive genetic testing without adequate support could lead to adverse psychological and behavioural responses to risk disclosure. Although the risk of psychological harm may in part be mitigated by self-selection into genetic testing[81], it will be vital to understand the origins of anticipated negative responses and provide adequate education and information about the nature of genetic testing before its integration into mainstream healthcare. This study provides evidence to suggest that before population genetic testing is made available, education is needed that is inclusive of people with different levels of information literacy. Further studies will be needed to evaluate education approaches and their impact on women's choices about whether to participate.

Reference List

1. Antoniou A, Easton DF. Average risks of breast and ovarian cancer associated with BRCA1 or BRCA2 mutations detected in case series unselected for family history: a combined analysis of 22 studies. American Journal of Human Genetics 2003;**72**(5):1117-30.
2. Evans DG, Shenton A, Woodward E, et al. Penetrance estimates for BRCA1 and BRCA2 based on genetic testing in a Clinical Cancer Genetics service setting: risks of breast/ovarian cancer quoted should reflect the cancer burden in the family. BMC Cancer 2008;**8**(1):155.
3. Ford D, Easton DF, Bishop DT, et al. Risks of cancer in BRCA1-mutation carriers. The Lancet 1994;**343**(8899):692-95.
4. Easton DF, Pooley KA, Dunning AM, et al. Genome-wide association study identifies novel breast cancer susceptibility loci. Nature 2007;**447**:1087-93.
5. Gayther SA, Warren W, Mazoyer S, et al. Germline mutations of the BRCA1 gene in breast and ovarian cancer families provide evidence for. Nature Genetics 1995;**11**(428-433).
6. Loveday C, Turnbull C, Ramsay E, et al. Germline mutations in RAD51D confer susceptibility to ovarian cancer. Nature Genetics 2011;**43**(9):879-82.
7. Meindl A, Hellebrand H, Wiek C, et al. Germline mutations in breast and ovarian cancer pedigrees establish RAD51C as a human cancer susceptibility gene. Nature Genetics 2010;**42**(5):410-14.
8. Song H, Dicks E, Ramus SJ, et al. Contribution of germline mutations in the RAD51B, RAD51C, and RAD51D genes to ovarian cancer in the population. Journal of Clinical Oncology 2015;**10**;33(26):2901-2907.
9. Ramus SJ, Song H, Dicks E, et al. Germline mutations in the BRIP1, BARD1, PALB2, and NBN genes in women with ovarian cancer. Journal of National Cancer Institute 2015;**107**(11)djv214.
10. Bowtell DD, Bohm S, Ahmed AA, et al. Rethinking ovarian cancer II: reducing mortality from high-grade serous ovarian cancer. Nat Rev Cancer 2015;**15**(11):668e79.
11. Antoniou AC, Casadei S, Heikkinen T, et al. Breast-cancer risk in families with mutations in PALB2. New England Journal of Medicine 2014;**371**(6):497-506.
12. Robson ME, Storm CD, Weitzel J, et al. American Society of Clinical Oncology policy statement update: genetic and genomic testing for cancer susceptibility. Journal of Clinical Oncology 2010;**28**(5):893-901.
13. Wacholder S, Hartge P, Prentice R, et al. Performance of common genetic variants in breast-cancer risk models. New England Journal of Medicine, 2010;**362**(11):986-93.
14. Stanislaw C, Xue Y, Wilcox WR. Genetic evaluation and testing for hereditary forms of cancer in the era of next-generation sequencing. Cancer Biology & Medicine 2016;**13**(1):55–67. http://doi.org/10.28092/j.issn.2095-3941.2016.0002
15. Manchanda R, Legood R, Burnell M, et al. Cost-effectiveness of population screening for BRCA mutations in Ashkenazi Jewish women compared with family history–based testing. Journal of the National Cancer Institute 2015;**107**(1):380.
16. Manchanda R, Loggenberg K, Gessler S, et al. Population based testing for high-penetrance dominant gene mutations: initial results from the pilot phase of GCaPPS. International Journal of Gynaecological Cancer 2012;**22**:S42-S43.
17. Gabai-Kapara E, Lahad A, Kaufman B, et al. Population-based screening for breast and ovarian cancer risk due to BRCA1 and BRCA2. Proceedings of the National Academy of Sciences 2014;**111**(39):14205-10.
18. King M, Levy-Lahad E, Lahad A. Population-based screening for brca1 and brca2: 2014 lasker award. Journal of the American Medical Association 2014;**312**(11):1091-92.
19. Levy-Lahad E, Lahad A, King MC. Precision Medicine Meets Public Health: Population Screening for BRCA1 and BRCA2. Journal of the National Cancer Institute 2015;**107**(1):420.
20. Slade I, Riddell D, Turnbull C, et al. Development of cancer genetic services in the UK: A national consultation. Genome medicine. 2015 Feb 2;7(1):18
21. Rahman, N. Mainstream genetic testing of cancer predisposition genes. Clinical Medicine (London, England), 2014;**14**(4):436–39.

For peer review only

22. Katsanis SH, Katsanis N. Molecular genetic testing and the future of clinical genomics. Nature Reviews. Genetics. 2013;**14**(6):415-26.
23. Chan IS, Ginsburg GS. Person-specificized medicine: progress and promise. Annual Review of Genomics and Human Genetics 2011;**29**(12):217-44.
24. Rapiti E, Pinaud K, Chappuis PO, et al. Opportunities for improving triple-negative breast cancer outcomes: results of a population-based study. Cancer Medicine. 2017;**6**(3):526-36.
25. Trainer AH, Lewis CR, Tucker K, et al. The role of BRCA mutation testing in determining breast cancer therapy. Nature reviews Clinical oncology. 2010;**7**(12):708-17.
26. Gray SW, O'Grady C, Karp L, et al. Risk information exposure and direct-to-consumer genetic testing for BRCA mutations among women with a person-specific or family history of breast or ovarian cancer. Cancer Epidemiology and Prevention Biomarkers. 2009;**18**(4):1303-11.
27. Annas GJ, Elias S. 23andMe and the FDA. New England Journal of Medicine 2014;**370**(11):985-88.
28. McGuire AL, Joffe S, Koenig BA, et al. Ethics and genomic incidental findings. Science 2013;**340**:1047-48.
29. Schuurman AG, van der Kolk DM, Verkerk MA, et al. Maximising the efficiency of clinical screening programmes: balancing predictive genetic testing with a right not to know. European Journal of Human Genetics 2015;**23**:1124-28.
30. Su Y, Borry P, Otte IC, et al. “It's our DNA, we deserve the right to test!” A content analysis of a petition for the right to access direct-to-consumer genetic testing. Personalized Medicine 2013;**10**(7):729-39.
31. Heshka JT, Palleschi C, Howley H, et al. A systematic review of perceived risks, psychological and behavioral impacts of genetic testing. Genetics in Medicine 2008;**10**(1):19-32.
32. Bosch N, Junyent N, Gadea N, et al. What factors may influence psychological well being at three months and one year post BRCA genetic result disclosure? The Breast 2012;**21**:755-60.
33. Ringwald J, Wochnowski C, Bosse K, et al. Psychological Distress, Anxiety, and Depression of Cancer-Affected BRCA1/2 Mutation Carriers: a Systematic Review. Journal of Genetic Counseling 2016;**25**:880-91.
34. Meiser B. Psychological impact of genetic testing for cancer susceptibility: An update of the literature. Psycho-Oncology 2005;**14**(12):1060-74.
35. Larouche G, Chiquette J, Pelletier S, et al. Do women change their breast cancer mammogram screening behaviour after BRCA1/2 testing? Familial Cancer 2016:1-6.
36. Beery TA, Williams JK. Risk reduction and health promotion behaviors following genetic testing for adult-onset disorders. Genetic Testing 2007;**11**(2):111-23.
37. Marteau TM, French DP, Griffin SJ, et al. Effects of communicating DNA-based disease risk estimates on risk-reducing behaviours. The Cochrane Library 2010.
38. Hollands GJ, French DP, Griffin SJ, et al. The impact of communicating genetic risks of disease on risk-reducing health behaviour: systematic review with meta-analysis. British Medical Journal 2016;**352**:i1102.
39. Biesecker BB, Ishibe N, Hadley DW, et al. Psychosocial factors predicting BRCA1/BRCA2 testing decisions in members of hereditary breast and ovarian cancer families. American Journal of Medical Genetics 2000;**93**(4):257-63.
40. Caulfield T, McGuire A, L. . Direct-to-consumer genetic testing: perceptions, problems, and policy responses. Annual Review of Medicine 2012;**63**:23-33.
41. Caulfield T, Ries NM, Ray PN, et al. Direct-to-consumer genetic testing: good, bad or benign? Clinical Genetics 2010;**77**(2):101-05.
42. Matloff E, Caplan A. Direct to confusion: lessons learned from marketing BRCA testing. The American Journal of Bioethics 2008;**8**(6):5-8.
43. George A, Kaye S, Banerjee S. Delivering widespread BRCA testing and PARP inhibition to patients with ovarian cancer. Nature Reviews Clinical Oncology. 2017;**14**(5):284-96.
44. Kentwell M, Dow E, Antill Y, et al. Mainstreaming cancer genetics: A model integrating germline BRCA testing into routine ovarian cancer clinics. Gynecologic Oncology. 2017;**145**(1):130-6.
45. George A, Riddell D, Seal S, et al. Implementing rapid, robust, cost-effective, patient-centred, routine genetic testing in ovarian cancer patients. Scientific reports. 2016; **13(**6):29506.

For peer review only

46. Plaskocinska I, Shipman H, Drummond J, et al. New paradigms for BRCA1/BRCA2 testing in women with ovarian cancer: results of the Genetic Testing in Epithelial Ovarian Cancer (GTEOC) study. Journal of Medical Genetics 2016.
47. Zilliacus EM, Meiser B, Lobb EA, et al. Are videoconferenced consultations as effective as face-to-face consultations for hereditary breast and ovarian cancer genetic counseling? Genetics in Medicine 2011;**13**(11):933-41.
48. Buchanan AH, Datta SK, Skinner CS, et al. Randomized trial of telegenetics vs. in-person cancer genetic counseling: Cost, patient satisfaction and attendance. Journal of Genetic Counseling 2015;**24**(6):961-70.
49. Meropol NJ, Daly MB, Vig HS, et al. Delivery of Internet-based cancer genetic counselling services to patients' homes: a feasibility study. Journal of Telemedicine and Telecare 2011;**17**:36–40.
50. Lerman C, Croyle RT, Tercyak KP, et al. Genetic testing: psychological aspects and implications. Journal of Consulting and Clinical Psychology 2002;**70**(3):784-97.
51. Ropka ME, Wenzel J, Phillips EK, et al. Uptake rates for breast cancer genetic testing: a systematic review. Cancer Epidemiology Biomarkers & Prevention 2006;**15**(5): 840-55.
52. Meisel SF, Side L, Fraser L, et al. Population-based, risk-stratified genetic testing for ovarian cancer risk: a focus group study. Public Health Genomics 2013;**16**(4):184-91.
53. Henneman L, Timmermans DR, Bouwman CM, et al. 'A low risk is still a risk': exploring women's attitudes towards genetic testing for breast cancer susceptibility in order to target disease prevention. Public Health Genomics 2011;**14**(4-5):238-47.
54. Ulrich CM, Kristal AR, White E, et al. Genetic testing for cancer risk: A population survey on attitudes and intention. Public Health Genomics 1998;**1**(14):213-22.
55. Henneman L, Vermeulen E, van El CG, et al. Public attitudes towards genetic testing revisited: comparing opinions between 2002 and 2010. European Journal of Human Genetics 2013;**21**(8):793-99.
56. Meisel SF, Pashayan N, Rahman B, et al. Adjusting the frequency of mammography screening on the basis of genetic risk: Attitudes among women in the UK. The Breast 2015;**24**(3):237-41.
57. Sanderson SC, Wardle J, Jarvis MJ, et al. Public interest in genetic testing for susceptibility to heart disease and cancer: a population-based survey in the UK. Preventive Medicine 2004;**39**(3):458-64.
58. Singer E. Public attitudes toward genetic testing. Population Research and Policy Review 1991;**10**(3):235-55.
59. Henneman L, Timmermans DRM, Wal GVD. Public Attitudes Toward Genetic Testing: Perceived Benefits and Objections. Genetic Testing 2006;**10**(2):139-45.
60. Gottfredson LS. Intelligence: is it the epidemiologists' elusive" fundamental cause" of social class inequalities in health? Journal of Personality and Social Psychology 2004;**86**(1).
61. Beeken RJ, Simon AE, von Wagner C, et al. Cancer fatalism: deterring early presentation and increasing social inequalities? Cancer Epidemiology Biomarkers & Prevention 2011;**20**(10):2127-31.
62. von Wagner C, Good A, Whitaker KL, et al. Psychosocial determinants of socioeconomic inequalities in cancer screening participation: a conceptual framework. Epidemiologic Reviews 2011;**33**(1):135-47.
63. Sanderson SC, Waller J, Jarvis MJ, et al. Awareness of lifestyle risk factors for cancer and heart disease among adults in the UK. Patient Education and Counseling 2009;**74**(2):221-27.
64. Kutner M, Greenberg E, Jin Y, et al. (2006) The Health Literacy of America's Adults: Results From the 2003 National Assessment of Adult Literacy (NCES 2006–483) U.S. Department of Education, Washington, DC: National Center for Education Statistics.
65. Lo SH, Waller J, Wardle J, et al. Comparing barriers to colorectal cancer screening with barriers to breast and cervical screening: a population-based survey of screening-age women in Great Britain. Journal of Medical Screening 2013;**20**(2):73-79.
66. Dent T, Jbilou J, Rafi I, et al. Stratified cancer screening: the practicalities of implementation. Public Health Genomics 2013;**16**(3):94-99.
67. Hall AE, Chowdhury S, Hallowell N, et al. Implementing risk-stratified screening for common cancers: a review of potential ethical, legal and social issues. Journal of Public Health 2014;**36**(2):285-91.
68. Lerman C, Daly M, Masny A, et al. Attitudes about genetic testing for breast-ovarian cancer susceptibility. Journal of Clinical Oncology 1994;**12**(4):843-50.

For peer review only

69. Bottorff JL, Ratner PA, Balneaves LG, et al. Women's interest in genetic testing for breast cancer risk the influence of sociodemographics and knowledge. Cancer Epidemiology Biomarkers & Prevention 2002;**11**(1):89-95.
70. Aro AR, Hakonen A, Hietala M, et al. Acceptance of genetic testing in a general population: age, education and gender differences. Patient Education and Counseling 1997;**32**(1):41-49.
71. Borzekowski DL, Guan Y, Smith KC, et al. The Angelina effect: immediate reach, grasp, and impact of going public. Genetics in Medicine 2013;**16**(7):516-21.
72. Kamenova K, Reshef A, Caulfield T. Angelina Jolie's faulty gene: newspaper coverage of a celebrity's preventive bilateral mastectomy in Canada, the United States, and the United Kingdom. Genetics in Medicine 2013;**16**:522-28.
73. Cameron LD, Reeve J. Risk perceptions, worry, and attitudes about genetic testing for breast cancer susceptibility. Psychology & Health 2006;**21**(2):211-30.
74. Lipkus IM, Klein WM, Skinner CS, et al. Breast cancer risk perceptions and breast cancer worry: What predicts what? Journal of Risk Research 2005;**8**(5):439-52.
75. Sheeran P, Webb TL. The Intention–Behavior Gap. Social and Personality Psychology Compass 2016;**10**(9):503-18.
76. Gilovich T, Griffin D, Kahneman D. *Heuristics and biases: The psychology of intuitive judgment*: Cambridge University Press, 2002.
77. Wilson TD, Gilbert DT. Affective forecasting. Advances in Experimental Social Psychology 2003;**35**:345-411.
78. Hann, KEJ, Freeman, M, Fraser, L, et al. Awareness, knowledge, perceptions, and attitudes towards genetic testing for cancer risk among ethnic minority groups: a systematic review. BMC Public Health 2017;17**:** 503.
79. Atkin, K, Ali N, Chu, CE. The politics of difference? Providing a cancer genetics service in a culturally and linguistically diverse society. Diversity in Health and Care 2009*;* 6**:** 149–157
80. Genetic Alliance UK. 2012. Identifying family risk of cancer: Why is this more difficult for ethnic minority communities and what would help? London.
81. Sanderson SC, Wardle J. Assosciations between anticipated reactions to genetic test results and interest in genetic testing: Will self-selection reduce the potential for harm? Genetic Testing 2008;**12**(1):59-66

For peer review only

Table 1: Sample demographic and personal characteristics (n=837)

	N (%)
**Gender**	
**Female**	837 (100.0)
**Age (mean, SD)**	45.9 (15.5)
**Age group**	
**18-29 years**	143 (17.1)
**30-39 years**	179 (21.4)
**40-49 years**	160 (19.1)
**50-64**	234 (28.0)
**65-74 years**	121 (14.5
**Ethnicity**	
**White British**	725 (86.6)
**White Other**	52 (6.3)
**Mixed ethnicity**	12 (1.4)
**Black**	11 (1.3)
**South Asian**	28 (3.3)
**Other**	9 (1.1)
**Education**	
**No formal qualifications**	126 (15.1)
**Less than degree level qualification**	478 (57.1)
**Degree level (or equivalent)**	233 (27.8)
**Marital status**	
**Married/ Civil partnership/ Cohabiting**	459 (54.8)
**Single**	177 (21.1)
**Divorced/ Separated**	140 (16.7)
**Widowed**	61 (7.3)
**Family history of cancer (mother, father, brother, sister)**	
**Yes**	305 (36.4)
**No/don't know**	532 (63.6)
**Perceived chances of getting ovarian cancer compared to other women of same age**	
**Much higher/higher**	64 (7.6)
**About the same as others**	590 (70.5)
**Much lower/lower**	183 (21.9)
**Perceived chances of getting breast cancer compared to other women of same age**	
**Much higher/higher**	83 (9.9)
**About the same as others**	616 (73.7)
**Much lower/lower**	137 (16.4)

For peer review only

Table 2. Univariate Analyses of anticipated reactions to risk disclosure of breast and ovarian cancer genetic risk

**Variable**	**'I would try harder to have a healthy lifestyle'[1]**		**'It would give me more control over my life'[1]**		**'I would feel that it wasn't worth making lifestyle changes'[1]**		**'I would feel less free to make choices in my life'[1]**	
	n (%) agree/ strongly agree	χ^2 (df) p-value	n (%) agree/ strongly agree	χ^2 (df) p-value	n (%) agree/ strongly agree	χ^2 (df) p-value	n (%) agree/ strongly agree	χ^2 (df) p-value
**Sample total (n = 837)**	611 (73.0)		452 (54.0)		119 (14.2)		143 (17.1)	
**Age**								
**>50**	248 (71.7)	0.52 (1)	199 (57.5)	2.92 (1)	54 (15.6)	0.93 (1)	55 (15.9)	0.58 (1)
**≤50**	363 (73.9)	0.469	253 (51.5)	0.087	65 (13.2)	0.334	88 (17.9)	0.443
**Ethnicity**								
**White**	567 (73.0)	0.004 (1)	415 (53.4)	1.52 (1)	111 (14.3)	0.04 (1)	**127 (16.3)**	**4.18 (1)**
**Ethnic minority**	44 (73.3)	0.952	37 (61.7)	0.218	8 (13.3)	0.839	**16 (26.7)**	**0.041***
**Education**								
**University degree**	162 (69.5)	1.97 (1)	**112 (48.1)**	**4.57 (1)**	**21 (9.0)**	**7.17 (1)**	34 (14.6)	1.41 (1)
**Below university degree**	449 (74.3)	0.160	**340 (56.3)**	**0.032***	**98 (16.2)**	**0.007***	119 (18.0)	0.234
**Marital status**								
**Married/cohabiting**	343 (75.4)	2.87 (1)	**261 (57.4)**	**4.53 (1)**	61 (13.4)	0.53 (1)	67 (14.7)	**3.91 (1)**
**Single/widowed/divorced**	268 (70.2)	0.090	**191 (50.0)**	**0.033***	58 (15.2)	0.463	76 (19.9)	**0.048***
**Family history of any cancer**								
**No/don't know**	385 (72.4)	0.29 (1)	**271 (50.9)**	**5.51 (1)**	73 (13.7)	0.29 (1)	98 (18.4)	1.84 (1)
**Yes**	226 (74.1)	0.587	**181 (59.3)**	**0.019***	46 (15.1)	0.588	45 (14.8)	0.175
**Perceived risk of OC (n = 847)**								
**Much lower than others**	40 (66.7)	6.92 (4)	30 (50.0)	4.08 (4)	11 (18.3)	1.26 (4)	**22 (36.7)**	**22.38 (4)**
**Lower than others**	94 (76.4)	0.140	66 (53.7)	0.394	17 (13.8)	0.867	**25 (20.3)**	**<0.001***
**The same as others**	427 (72.4)		314 (53.2)		83 (14.1)		**84 (14.2)**	
**Higher than others**	48 (81.4)		39 (66.1)		7 (11.9)		**10 (16.9)**	
**Much higher than others**	2 (40.0)		3 (60.0)		1 (20.0)		**2 (40.0)**	

For peer review only

**Perceived risk of BC (n = 847)**								
**Much lower than others**	16 (64.0)	3.58 (4)	8 (32.0)	5.17 (4)	6 (24.0)	3.46 (4)	**8 (32.0)**	**11.08 (4)**
**Lower than others**	88 (78.6)	0.466	61 (54.5)	0.269	14 (12.5)	0.484	**27 (24.1)**	**0.026***
**The same as others**	444 (72.0)		337 (54.6)		84 (13.6)		**92 (14.9)**	
**Higher than others**	55 (75.3)		41 (56.2)		13 (17.8)		**13 (17.8)**	
**Much higher than others**	8 (80.0)		5 (50.0)		2 (20.0)		**3 (30.3)**	

[1]Each statement preceded by: If I knew my risk of breast and ovarian cancer, I would…; Abbreviations: BC = breast cancer, OC = ovarian cancer: * Significant at p <0.05

For peer review only

Table 3. Multivariate logistic regression of demographic and person-specific predictors of anticipated reactions to disclosure of genetic risk of breast and ovarian cancer

Variable (n = 837)	‘I would try harder to have a healthy lifestyle’[1]			It would give me more control over my life’[1]			‘I would feel it wasn’t worth making lifestyle changes’[1]			‘I would feel less free to make choices in my life’[1]		
	OR	95% CI	p-value	OR	95% CI	p-value	OR	95% CI	p-value	OR	95% CI	p-value
**Age**												
>50	1			1			1			1		
≤50	1.86	0.84-1.66	0.321	0.80	0.60-1.11	0.205	0.99	0.64-1.53	0.988	1.22	0.81-1.85	0.333
**Ethnicity**												
White	1			1			1			1		
Ethnic minority	0.95	0.51-1.66	0.859	1.51	0.86-2.64	0.149	1.01	0.45-2.22	0.985	1.60	0.86-3.01	0.138
**Education**												
University degree	1			1			**1**			1		
Below university degree	1.35	0.96-1.91	0.081	1.39	1.01-1.91	0.039	**1.99**	**1.19-3.35**	**0.009***	1.32	0.85-2.03	0.333
**Marital status**												
Single/widowed/divorced	1			1			1			1		
Married/cohabiting	1.34	0.98-1.83	0.062	1.41	1.07-1.87	0.016	0.92	0.62-1.38	0.704	0.69	0.50-1.01	0.051
**Family history of any cancer**												
No/don’t know	1			1			1			1		
Yes	1.14	0.81-1.61	0.438	1.31	0.96-1.78	0.085	1.09	0.70-1.68	0.695	0.81	0.54-1.24	0.345
**Perceived risk of OC**												
Much lower/lower than others	1			1			1			1		
The same as others	1.15	0.68-1.95	0.588	0.95	0.58-1.55	0.848	0.90	0.47-1.72	0.759	0.55	0.30-0.99	0.046
Higher/much higher than others	1.41	0.64-3.15	0.392	1.86	0.90-3.84	0.092	0.54	0.19-1.51	0.241	0.61	0.25-1.48	0.276
**Perceived risk of BC**												
Much lower/lower than others	1			1			1			1		
The same as others	0.65	0.36-1.19	0.171	1.29	0.75-2.22	0.349	0.95	0.46-1.99	0.899	0.81	0.43-1.55	0.538
Higher/much higher than others	0.70	0.32-1.54	0.376	0.97	0.49-1.94	0.934	1.44	0.57-3.63	0.431	0.99	0.43-2.33	0.996

[1]Each statement was preceded by: If I knew my risk of breast and ovarian cancer…

Abbreviations: BC = breast cancer, OC = ovarian cancer

* Significant after Bonferroni correction at p <0.012

For peer review only

Supplementary Table 1. Full set of responses for outcome variables

If I knew my risk of breast and ovarian cancer…	N (%)
**I would try harder to have a healthy lifestyle**	
Strongly disagree	33 (3.9)
Disagree	50 (6.0)
Neither agree/disagree	143 (17.1)
Agree	425 (50.8)
Strongly agree	186 (22.2)
**It would give me more control over my life**	
Strongly disagree	22 (2.6)
Disagree	135 (16.1)
Neither agree/disagree	228 (27.2)
Agree	387 (46.2)
Strongly agree	65 (7.8)
**I would feel it wasn't worth making lifestyle changes**	
Strongly disagree	169 (20.2)
Disagree	397 (47.4)
Neither agree/disagree	152 (18.2)
Agree	104 (12.4)
Strongly agree	15 (1.8)
**I would feel less free to make choices in my life**	
Strongly disagree	112 (13.4)
Disagree	402 (48.0)
Neither agree/disagree	180 (21.5)
Agree	129 (15.4)
Strongly agree	14 (1.7)

For peer review only

BMJ Open

Anticipated health behaviour changes and perceived control in response to disclosure of genetic risk of breast and ovarian cancer: a quantitative survey study among women in the UK

Journal:	*BMJ Open*
Manuscript ID	bmjopen-2017-017675.R2
Article Type:	Research
Date Submitted by the Author:	02-Oct-2017
Complete List of Authors:	Meisel, Susanne; Kings College London<br>Fraser, Lindsay; Institute for Women's Health, Department of Women's Cancer<br>Side, Lucy; University College London Institute for Women's Health<br>Gessler, Sue; University College London Hospitals NHS Foundation Trust, ; UCL Institute for Women's Health and NIHR University College London Hospitals Biomedical Research Centre, Department of Women's Cancer<br>Hann, Katie; University College London Institute for Women's Health, Department of Women's Cancer<br>Wardle, Jane; University College London, Dept. of Epidemiol<br>Lanceley, Anne; UCL, Department of Women's Cancer
**Primary Subject Heading**:	Genetics and genomics
Secondary Subject Heading:	Public health
Keywords:	Breast cancer, Ovarian cancer, Predictive genetic testing, Risk

SCHOLARONE™
Manuscripts

For peer review only

TITLE: Anticipated health behaviour changes and perceived control in response to disclosure of genetic risk of breast and ovarian cancer: a quantitative survey study among women in the UK

Authors: Susanne F Meisel PhD[1], Lindsay Fraser BSc[2], Lucy Side MD[2], Sue Gessler PhD[2], Katie E J Hann MSc[2], Jane Wardle PhD[3]† and Anne Lanceley PhD[2] for the PROMISE study team

[1]Institute of Psychiatry, Psychology and Neuroscience, King's College London, London, UK.

[2]Department of Women's Cancer, EGA UCL Institute for Women's Health, University College London, London, UK.

[3]Department of Behavioural Science and Health, Institute of Epidemiology and Health Care, University College London, London, UK.

Corresponding Author:

Anne Lanceley, PhD

237c

Medical School Building

74 Huntley Street

London, WC1E 6AU

Email: a.lanceley@ucl.ac.uk

Phone: +4420 7679 6807

For peer review only

Abstract

Background: Genetic risk assessment for breast cancer and ovarian cancer (BCOC) is expected to make major inroads into mainstream clinical practice. It is important to evaluate the potential impact on women ahead of its implementation in order to maximise health benefits, as predictive genetic testing without adequate support could lead to adverse psychological and behavioural responses to risk disclosure.

Objective: To examine anticipated health behaviour changes and perceived control to disclosure of genetic risk for BCOC, and establish demographic and person-specific correlates of adverse anticipated responses in a population-based sample of women.

Design: Cross-sectional quantitative survey study carried out by the UK Office for National Statistics in January and March 2014.

Setting: Face-to-face computer-assisted interviews conducted by trained researchers in participants' homes.

Participants: 837 women randomly chosen from households across the UK identified from the Royal Mail's Postcode Address File.

Outcome measures: Anticipated health behaviour change and perceived control to disclosure of BCOC risk.

Results: In response to a genetic test result, most women (72%) indicated 'I would try harder to have a healthy lifestyle' and over half (55%) felt 'it would give me more control over my life'. These associations were independent of demographic factors or perceived risk of BCOC in Bonferroni-corrected multivariate analyses. However, a minority of women (14%) felt 'it isn't worth making lifestyle changes', and that 'I would feel less free to make choices in my life' (16%) in response to BCOC risk disclosure. The former belief was more

For peer review only

likely to be held by women who were educated below university degree level (p <0.001) after adjusting for other demographic and person-specific correlates.

Conclusion: These findings indicate that women in the UK largely anticipate that they would engage in positive health behaviour changes in response to BCOC risk disclosure.

Strengths and limitations of this study

- Stratified random sampling, the 'gold-standard' for survey recruitment, was used in this study.
- The study reports on anticipated responses to genetic BCOC risk information from a large general population sample of women living in the UK.
- The survey questions were broad, and may have been interpreted differently by different participants
- A general hypothetical scenario about receiving genetic BCOC risk information was used instead of asking participants to consider a hypothetical scenario of receiving a high or low risk genetic test result.

For peer review only

Source of funding:

This research was funded by grants from The Eve Appeal (London, UK, grant code: 509050) and Cancer Research UK (London, UK; grant code: 508007). Jane Wardle was supported by Cancer Research UK as part of the CR UK-UCL Cancer Centre.

Conflicts of interest:

All authors have completed the ICMJE uniform disclosure form at www.icmje.org/coi_disclosure.pdf and declare: SFM and KEJH had financial support from Cancer Research UK and the Eve Appeal for the submitted work; no financial relationships with any organisations that might have an interest in the submitted work in the previous three years; no other relationships or activities that could appear to have influenced the submitted work.

Authors contributions:

SFM participated in study design, analysed the data and drafted the manuscript; KEJH helped with data interpretation and critically revised the manuscript; LS, LF, SG, AL and JW conceived the study, participated in study design and coordination and critically revised the manuscript. All authors have seen and approved the final manuscript except JW who sadly died before submission of the manuscript.

Data sharing statement

The data is available on request from the corresponding author: a.lanceley@ucl.ac.uk

Word count: 4014

Number of tables: 3 main and 1 supplementary

For peer review only

Introduction

Advances in genetic technologies have led to the identification of gene mutations associated with an increased risk of developing breast cancer and ovarian cancer (BCOC). In addition to germline mutations in *BRCA 1* and *BRCA 2*, which confer a very high lifetime risk of BCOC (39%- 65% and 11%-37%, respectively) [1-3], a growing list of moderate- and lower-risk markers specific to each cancer type have been identified [4-10]. These influence cancer development in a larger proportion of the population despite small effect sizes. It is anticipated that, in future, some moderate risk markers will be included in existing panel tests used in clinical genetics practice[11-13]. While high penetrance genes like *BRCA1* and *BRCA2* confer a lifetime risk for cancer of 50% or greater, the lifetime risk for moderately penetrant genes ranges from 20% to 50% and low penetrance genes are thought to have a limited effect[14].

To date, genetic testing of *BRCA1* and *BRCA2* is conducted in clinical practice only, in individuals with a strong family history, or after a cancer diagnosis. However, it has been argued that this approach is likely to miss a substantial proportion of women at high risk (~30%), particularly if the family is small, the mutation was transmitted through the paternal line, or the individual has a number of lower risk variants whose effects are additive[15-18]. Given that genetic testing for BCOC risk can now be carried out at a relatively low cost, there have been calls to make it available on a population level, based on the premise that timely awareness of genetic risk would empower individuals to make informed decisions about cancer risk management, allow for early intervention, and ultimately, improve clinical outcomes[19]. There is a drive from healthcare professionals to 'mainstream' genetic testing as part of routine cancer care in oncology services, aimed at improving diagnosis and providing individualised treatment for patients (http://www.mcgprogramme.com/)[20-23]. *BRCA1* and *BRCA2* testing may be offered to women diagnosed with triple negative breast cancer and information on genetic status is likely to influence clinical care and management of the patient and the family[24-25]. Moreover, genetic testing for BCOC susceptibility can

already be purchased over the Internet[26] (www.23andme.com; www.getcolor.com). The notion that individuals have a 'right to know' their genetic information has also gained traction in recent years[27-30].

However, concerns persist about patient safety related to poor psychological adjustment following genetic risk disclosure. Studies investigating reactions to disclosure of BRCA status commonly do not find evidence for persistent negative psychological outcomes beyond one year after result disclosure[31-34]. There is also some evidence suggesting that genetic test results indicating a higher risk of disease can lead to behaviour change such as uptake of breast and colorectal cancer screening[31 35 36], although two systematic reviews found no significant impact of genetic risk information on other behaviours[37 38] including smoking, physical activity, use of medication or vitamins, diet, alcohol use and use of a sunscreen. However, genetic testing has hitherto only been performed in women with a strong family history of BCOC, so these results may not generalise to a population largely unaware of any increased risk.

Currently, one-to-one genetic counselling is strongly embedded within clinical genetic testing services, offering education and support throughout the process. If genetic testing were introduced on a population level, the current model of delivering genetic counselling would no longer be feasible. It has been argued that returning genetic risk test results for highly debilitating conditions such as BCOC without appropriate professional guidance would leave room for misinterpretation and uncertainty, increasing the risk of adverse psychological outcomes[39-42]. In recent years, research has begun to investigate new approaches of providing cancer genetic services. Mainstreaming genetic testing, by offering women with a diagnosis of ovarian cancer testing directly through the oncology clinic and providing post-test genetic counselling via clinical genetics for those with a pathogenic

For peer review only

variant, is one example[43-44]. Research has found that mainstreaming genetic testing in this way can successfully reduce time and resources spent on the processes[45] and does not appear to negatively affect patients' psychological well-being[46]. Genetic counselling with patients and healthy family members via telephone or video consultations instead of face-to-face genetic counselling has also been found to be feasible and patients are generally satisfied[47-49]. These new approaches to offering genetic testing and providing genetic counselling for cancer susceptibility may be advantageous in future if testing is offered to patients more widely.

Although a wealth of studies have investigated anticipated psychological reactions to disclosure of BCOC genetic risk in higher risk groups[31 50 51] little is known about this issue in women unselected for family history. One qualitative study found that women in the UK would support the idea of genetic testing for risk of ovarian cancer because they expected it to trigger positive lifestyle changes and provide a means of control over cancer development[52]. However, in a similar study investigating attitudes towards genetic testing for breast cancer risk in the Netherlands, women were more ambivalent about disclosure of genetic risk; although there was recognition of the benefits of testing, some women were uncertain about their reactions to risk disclosure and the impact it would have on their lives[53].

An early population-based survey investigated attitudes towards genetic testing for breast cancer among 836 women living in Washington State[54]. Results showed that over three quarters of the sample expected that awareness of their genetic risk of breast cancer would give them more control over their life, and a similar number reported that they would 'definitely' or 'probably' take part in testing if it was offered to them. A more recent

For peer review only

population-based study with over 800 participants in the Netherlands compared attitudes towards genetic testing between 2002 and 2010[55]. Results showed that positive expectations about the utility of genetic testing had increased over the 8-year interval. However, about a third of participants believed that awareness of genetic risk would deprive people of the freedom to live as they want, and this proportion remained unchanged over time. Although these findings give an indication about the acceptability of population-based genetic testing, they may be culture-specific and may therefore not generalise to other populations. Furthermore, the latter survey asked only about attitudes towards genetic testing more broadly; attitudes to genetic risk disclosure specific to BCOC may be different.

The aim of the present study was to examine the anticipated health behaviour changes and perceived control in response to disclosure of genetic risk for BCOC and to explore demographic and person-specific correlates (for example marital status and family history of cancer) of anticipated response among a population sample of women in the UK. Identifying subgroups who are more likely to anticipate a negative response to learning about their genetic risk of developing these cancers, before genetic testing is integrated into mainstream healthcare (e.g. by including it in current cancer screening programmes), gives scope for timely intervention to avoid further increasing disparities in healthcare.

Methods and Procedure

This quantitative study complied with the principles set out in the declaration of Helsinki. As a population-based anonymous survey, it was exempt from ethical approval, in accordance with the guidelines set out by the UCL Ethics committee for non-NHS research (http://ethics.grad.ucl.ac.uk/exemptions.php).

Sample

Data were collected by the UK Office for National Statistics (ONS) in their monthly survey which is conducted on behalf of a range of organisations (e.g. government departments, non-governmental institutions and academic departments). Each month, 2010 households are identified from the Royal Mail's Postcode Address File using stratified random probability sampling. Selected addresses are contacted up to eight times at different times and days of the week to maximize response rates. One person aged over 16 from each household is randomly chosen to complete a computer-assisted face-to-face interview with trained researchers. Surveys commonly include a variety of different topics, making participation bias unlikely, although participants can opt out at any point. Questions on the 'genetics and screening' module were included in data collection waves in January and March 2014.

Measures

The 'genes and cancer' module was introduced with a short statement: '*Genes contain the 'instruction manual' of life, called DNA. Genes are passed from parents to their children. Nowadays, it is possible to predict whether someone is likely to develop certain diseases by looking at their genes. This is called genetic testing'*. No further information was given, as we were interested in women's attitudes towards BCOC genetic testing based on their current understanding of it. Participants were asked several questions in relation to genetic testing for BCOC risk and attitudes on breast cancer screening (see supplementary material for detail). Some of the findings have previously been published[56].

Outcome variables

Anticipated reactions to learning about increased BCOC risk were assessed with statements ranging from positively framed outcomes such as the opportunity to exert personal control ('If I knew my genetic risk of breast cancer and ovarian cancer, it would give me more control over my life') or to pursue health behaviours aimed at mitigating risk ('If I knew my genetic risk of breast cancer and ovarian cancer I would try harder to have a healthy lifestyle'), to more negatively framed outcomes such as curtailing freedom of choice ('If I knew my genetic risk of breast cancer and ovarian cancer, I would feel less free to make choices in my life') or the perceived futility of attempting behaviour change ('If I knew my genetic risk of breast cancer and ovarian cancer, I would feel it wasn't worth making lifestyle changes'). All items were scored on a five-point Likert scale ranging from 'strongly disagree'- 'strongly agree', and were adapted from previous research[54 55].

Predictor variables

Perceived risk of BCOC was each assessed with one question 'Compared with other women of your age, what do you think are your chances of getting breast [ovarian] cancer' with response options of: 'much lower than others', 'lower than others', 'the same as others', 'higher than others', 'much higher than others'. Given that individual risk categories pertaining to much lower/much higher perceived risk were small, perceived risk was grouped into three categories: 'much lower/lower than others; 'same as others', and 'higher/much higher than others'.

Age, ethnicity, educational attainment and marital status were derived using standard survey items (https://tinyurl.com/ya6gn2g6). Age was coded as ≤50 vs. >50 years to investigate any generational differences in anticipated reactions and attitudes. We chose 50

For peer review only

years of age as the cut-off point since public interest in the UK for genetic testing has been reported as being greatest for people aged 46-60 years[57] and we felt that the age of 50, being close to the average age of menopause in the UK (which is 51), was a reasonable mid-point to assess generational effects. Ethnicity was classified as 'White' vs. 'ethnic minority' because the individual ethnic minority sub-groups were small. Educational attainment was classified as university degree or equivalent vs. below university degree level. Marital status was coded as married/cohabiting vs. single/widowed/divorced. Family history of any cancer was assessed with one question: 'Have your mother, father, or any of your brothers or sisters, been diagnosed with cancer?' with response options being 'yes', 'no', and 'don't know'.

Statistical analyses

We included women aged 18-74 to reflect the population to whom genetic testing for risk of BCOC would likely become available in the future. Statistical analyses were carried out using the Statistical Package for Social Sciences (SPSS) version 20.0 (SPSS Inc., Chicago, IL). Descriptive statistics were explored using frequency tables. We explored demographic (age, ethnicity, education) and person-specific predictors (marital status, family history of cancer, perceived risk of ovarian cancer, perceived risk of breast cancer) for each outcome variable using χ^2 tests. Multivariate logistic regression models were used to investigate the demographic and person-specific correlates of anticipated reactions to disclosure of personal BCOC risk. Bonferroni corrections were employed in all multivariate analyses to correct for multiple testing, with $\alpha = 0.0125$.

For peer review only

Results

Demographic and personal characteristics

Data for the current study were collected in two waves: January and March 2014. In the January wave, 8% (n = 166) of the 2,010 selected households were not eligible because they were businesses or empty properties. Of 1,844 eligible households, 9% (n = 171) could not be contacted and 33% (n = 608) declined to take part in the ONS survey. In the March wave, 1,853 households were eligible (92%). Of those, 13% (n = 237) could not be contacted and 31% (n = 578) chose not to take part. Therefore, the overall response rate was 57%, comparable to previous ONS surveys (ONS 2013; ONS 2012; http://www.ons.gov.uk/ons/index.html). The total female sample was n=1,095.

After excluding participants with incomplete data for any of the outcome variables (n= 120), or who were aged younger than 18 or older than 74 years (n = 138), the final sample for analysis consisted of 837 women. Table 1 presents the demographic and personal characteristics of the sample. In common with other survey studies, education was higher in the sample than in the general population; other demographic characteristics were comparable to the UK population of women aged 18-74 (ONS Census 2011: (http://www.ons.gov.uk/ons/guide-method/census/2011/index).

Although 36.4% (n = 305) of the sample reported a family history of any cancer, most women felt that they were not particularly at risk of breast or ovarian cancer; only 9.9% (n = 83) thought they were at ‘higher’ or ‘much higher’ than average risk of breast cancer, and even fewer (7.6%, n = 64) thought they were at ‘higher’ or ‘much higher’ risk of ovarian cancer. A full breakdown of responses can be found in the supplementary material Table 1.

For peer review only

Support of genetic testing

Overall, women were supportive of the idea of genetic testing for breast and ovarian cancer. Only 2.0% (n = 18) thought that it was 'never' a good time to have a genetic test. However, about a third of the sample (31.4%, n = 290) were 'unsure'. Opinions were divided on when the best time would be to get tested, with 8.3% (n = 77) thinking that it would be 'just after birth', just over a quarter (27.4%, n = 253) thought 'during school', 10.6% (n = 98) reported 'just before marriage', 16.9% (n=156) 'before they plan to have a child', and 3.4% (n = 31) thought the best time would be 'when they are unwell'. There were no demographic or personal correlates with 'ever' vs. 'never' supporting genetic testing for breast and ovarian cancer (data not shown).

Anticipated response to disclosure of genetic risk of breast and ovarian cancer

Overall, women anticipated to respond proactively to awareness of their genetic risk. Most (71.9%, n = 669) said that they 'would try harder to have a healthy lifestyle', and over half (54.7%, n = 506) anticipated that it would give them 'more control over my life'. However, a minority reported that it would 'not be worth making lifestyle changes' (14.1%, n = 130) in response to genetic risk disclosure, and that they would feel 'less free to make choices in my life' (16.4%, n = 152).

In univariate analyses, there were no differences across demographic and person-specific variables in the expectation that disclosure of genetic risk of BCOC would lead to increased personal efforts 'to have a healthy lifestyle' (Table 2). Women who had fewer years of formal education (χ^2 (1) = 4.57, p = 0.032), were married (χ^2 (1) = 4.53, p = 0.033), or had a family history of cancer (χ^2 (1) = 5.51, p = 0.019) were significantly more likely to report that

For peer review only

awareness of genetic risk would give them more control over their life (Table 2). However, none of these associations remained after adjustment for covariates and multiple testing (Table 3).

Women with fewer years of formal education were also significantly more likely to agree that 'it wouldn't be worth making lifestyle changes' in response to genetic risk disclosure (χ^2 (1) = 7.17, p = 0.007), and this association remained in multivariate analyses (OR = 1.99, 95% CI = 1.19-3.35, p = 0.009). Women from ethnic minority backgrounds (χ^2 (1) = 4.18, p = 0.041), those who perceived themselves at a (much) lower or (much) higher risk of breast cancer (χ^2 (4) = 11.08, p = 0.026) or ovarian cancer (χ^2 (4) = 22.38, p<0.001) were more likely to report that awareness of genetic risk would make them 'feel less free to make choices in their life', but none of these findings was maintained after adjusting for demographic and person-specific factors, and for multiple testing (Table 3).

Discussion

This study investigated demographic and person-specific correlates of anticipated health behaviour changes and perceived control in response to disclosure of genetic risk of BCOC in a population-based sample of women in the UK.

Although our results suggested that most women did not expect to be negatively affected by knowing their genetic risk status for BCOC, women who had fewer years of formal education were nearly twice more likely to believe that 'it would not be worth making lifestyle changes' than those who had stayed in formal education for longer. This finding supports some earlier studies into genetic determinism (the idea that genetic make-up is

For peer review only

more important than environmental impact for expression of diseases)[58]; although not all studies have found this link[54 59].

However, the finding that women with fewer years of formal education are less likely to believe that lifestyle changes could be effective for cancer prevention also echoes studies that investigated attitudes towards cancer screening. These studies have found that women with lower socio-economic status, which is linked to educational attainment and information literacy[60], were more likely to hold fatalistic attitudes towards cancer development. Fatalistic attitudes have in turn been implicated in lower levels of engagement with cancer screening[61-62]. It is possible that women who do not believe in the curability of cancer *per se* are also more likely to report that 'it wouldn't be worth making lifestyle changes' in response to disclosure of BCOC genetic risk, but because we did not assess beliefs about curability of BCOC, this hypothesis remains speculative. Furthermore, research has shown that those with fewer years in education are less aware of cancer risk factors such as poor diet and physical inactivity[63]. A lack of awareness that lifestyle affects a person's risk of cancer would also help to explain why some participants may feel that 'it wouldn't be worth making lifestyle changes'. Although formal years of education do not equate with health literacy, which was not measured in this study, educational attainment is strongly associated with health literacy[64], and can therefore serve as an indicator for health literacy. Given that there are already disparities in engagement with available means of early detection for cancer (e.g. screening) across the population, with lower socioeconomic status groups less likely to engage[65], it will be vital to provide adequate education and information about the nature of genetic testing before it is introduced into mainstream healthcare. This point has also been highlighted by other commentators in the field[66-67]. In

For peer review only

addition, it is important that the public has a multifaceted view of cancer and are aware of genetic and non-genetic risk factors and ways to reduce risk despite genetic susceptibility.

Although previous studies in both higher-risk[68-69] and population samples[52 70] have shown that younger women are generally more positive towards genetic testing, we found no age differences in anticipated reactions towards BCOC genetic risk disclosure. It is interesting to speculate about the impact on our study respondents about the benefits of genetic testing as a result of the spike in media coverage that took place shortly before the survey responses were collected at the beginning of 2014. The well-known actress Angelina Jolie publicly announced her reasons for undergoing BRCA genetic testing and talked about how the knowledge that she carried a gene variant had enabled her to choose risk-reducing surgery (double mastectomy) to manage her increased risk of developing breast cancer. This celebrity revelation may have raised awareness of the potential benefits of breast (and ovarian) cancer genetic testing across all age groups[71-72], which may at least partially account for the finding that older women were as positive about genetic testing as younger women.

We found no association between participants' anticipated response to genetic risk disclosure and perceived risk of either breast or ovarian cancer. However, since few women considered themselves to be at 'higher' or 'much higher' than average risk of BCOC, sample sizes may have been too small to detect associations. Alternatively, these findings may suggest that UK women may have a more realistic understanding of their personal risk of BCOC compared to reports in previous studies from other countries[73-74]. Given that all survey items were closed questions, we could not investigate this finding in more depth, but this could be done in future research.

For peer review only

The strengths of this study are the approach to sampling (the sample was selected using stratified random sampling which is the gold-standard of survey recruitment and therefore, self-selection is unlikely to have biased current findings) and the statistical adjustment for multiple testing, which gives confidence in the robustness of the findings. However, the study also had important limitations. We only assessed anticipated responses to disclosure of genetic risk of BCOC in general, not whether these differed in response to a hypothetical 'higher' or 'lower' genetic risk. Although all questions have been used in previous research with different samples, with comparable findings, anticipated lifestyle changes and their perceived effectiveness, could vary depending on the levels of risk conferred. Further research could explore whether attitudes to genetic risk disclosure of BCOC differ when considering different levels of risk. Secondly, because of survey constraints, we focused our investigation on a limited set of broad questions which could have been interpreted differently by participants. Questions therefore remain about exactly how respondents anticipated changing their behaviour. Future research could investigate this topic with more in-depth questions.

Naturally, responses to 'real' disclosure of genetic risk information may be less positive than our results suggest; while most participants in this study indicated anticipated making an effort to 'have a healthy lifestyle', research has shown that there is generally a 'gap' between intentions and actual behaviour[75]. In addition, given that many individuals have difficulty in accurately anticipating emotional reactions to life events[76-77], these findings can only give a broad indication about potential effects of disclosure of BCOC genetic risk. Since none of the questions were open ended, we could not explore the origins of responses in more detail, but this could be done in future research. Finally, although ethnic distribution

of the sample was in line with the overall population in the UK, because ethnic minority subgroups were small we were unable to conduct more detailed analyses to investigate whether attitudes to genetic testing differed among women of different ethnic minority backgrounds. It will be important to explore this in future research, particularly since a recent review found a significant lack of research investigating UK black and minority ethnic (BAME) group awareness and attitudes toward genetic testing for cancer risk[78]. Reports that have involved UK BAME groups highlight low awareness of the availability of genetic services and fear and stigma in relation to cancer genetics[79-80].

Overall, findings from this study indicate that women in the UK are generally positive about the prospect of population based genetic testing for risk of BCOC. However, those with fewer years in education may be less positive. Given that there are already existing disparities in uptake of available means of early detection for breast cancer, careful planning is needed to avoid increasing these further. There are concerns in professional circles that predictive genetic testing without adequate support could lead to adverse psychological and behavioural responses to risk disclosure. Although the risk of psychological harm may in part be mitigated by self-selection into genetic testing[81], it will be vital to understand the origins of anticipated negative responses and provide adequate education and information about the nature of genetic testing before its integration into mainstream healthcare. This study provides evidence to suggest that before population genetic testing is made available, education is needed that is inclusive of people with different levels of information literacy. Further studies will be needed to evaluate education approaches and their impact on women's choices about whether to participate.

Reference List

1. Antoniou A, Easton DF. Average risks of breast and ovarian cancer associated with BRCA1 or BRCA2 mutations detected in case series unselected for family history: a combined analysis of 22 studies. American Journal of Human Genetics 2003;**72**(5):1117-30.
2. Evans DG, Shenton A, Woodward E, et al. Penetrance estimates for BRCA1 and BRCA2 based on genetic testing in a Clinical Cancer Genetics service setting: risks of breast/ovarian cancer quoted should reflect the cancer burden in the family. BMC Cancer 2008;**8**(1):155.
3. Ford D, Easton DF, Bishop DT, et al. Risks of cancer in BRCA1-mutation carriers. The Lancet 1994;**343**(8899):692-95.
4. Easton DF, Pooley KA, Dunning AM, et al. Genome-wide association study identifies novel breast cancer susceptibility loci. Nature 2007;**447**:1087-93.
5. Gayther SA, Warren W, Mazoyer S, et al. Germline mutations of the BRCA1 gene in breast and ovarian cancer families provide evidence for. Nature Genetics 1995;**11**(428-433).
6. Loveday C, Turnbull C, Ramsay E, et al. Germline mutations in RAD51D confer susceptibility to ovarian cancer. Nature Genetics 2011;**43**(9):879-82.
7. Meindl A, Hellebrand H, Wiek C, et al. Germline mutations in breast and ovarian cancer pedigrees establish RAD51C as a human cancer susceptibility gene. Nature Genetics 2010;**42**(5):410-14.
8. Song H, Dicks E, Ramus SJ, et al. Contribution of germline mutations in the RAD51B, RAD51C, and RAD51D genes to ovarian cancer in the population. Journal of Clinical Oncology 2015;**10**;33(26):2901-2907.
9. Ramus SJ, Song H, Dicks E, et al. Germline mutations in the BRIP1, BARD1, PALB2, and NBN genes in women with ovarian cancer. Journal of National Cancer Institute 2015;**107**(11)djv214.
10. Bowtell DD, Bohm S, Ahmed AA, et al. Rethinking ovarian cancer II: reducing mortality from high-grade serous ovarian cancer. Nat Rev Cancer 2015;**15**(11):668e79.
11. Antoniou AC, Casadei S, Heikkinen T, et al. Breast-cancer risk in families with mutations in PALB2. New England Journal of Medicine 2014;**371**(6):497-506.
12. Robson ME, Storm CD, Weitzel J, et al. American Society of Clinical Oncology policy statement update: genetic and genomic testing for cancer susceptibility. Journal of Clinical Oncology 2010;**28**(5):893-901.
13. Wacholder S, Hartge P, Prentice R, et al. Performance of common genetic variants in breast-cancer risk models. New England Journal of Medicine, 2010;**362**(11):986-93.
14. Stanislaw C, Xue Y, Wilcox WR. Genetic evaluation and testing for hereditary forms of cancer in the era of next-generation sequencing. Cancer Biology & Medicine 2016;**13**(1):55–67. http://doi.org/10.28092/j.issn.2095-3941.2016.0002
15. Manchanda R, Legood R, Burnell M, et al. Cost-effectiveness of population screening for BRCA mutations in Ashkenazi Jewish women compared with family history–based testing. Journal of the National Cancer Institute 2015;**107**(1):380.
16. Manchanda R, Loggenberg K, Gessler S, et al. Population based testing for high-penetrance dominant gene mutations: initial results from the pilot phase of GCaPPS. International Journal of Gynaecological Cancer 2012;**22**:S42-S43.
17. Gabai-Kapara E, Lahad A, Kaufman B, et al. Population-based screening for breast and ovarian cancer risk due to BRCA1 and BRCA2. Proceedings of the National Academy of Sciences 2014;**111**(39):14205-10.
18. King M, Levy-Lahad E, Lahad A. Population-based screening for brca1 and brca2: 2014 lasker award. Journal of the American Medical Association 2014;**312**(11):1091-92.
19. Levy-Lahad E, Lahad A, King MC. Precision Medicine Meets Public Health: Population Screening for BRCA1 and BRCA2. Journal of the National Cancer Institute 2015;**107**(1):420.
20. Slade I, Riddell D, Turnbull C, et al. Development of cancer genetic services in the UK: A national consultation. Genome medicine. 2015 Feb 2;7(1):18
21. Rahman, N. Mainstream genetic testing of cancer predisposition genes. Clinical Medicine (London, England), 2014;**14**(4):436–39.
22. Katsanis SH, Katsanis N. Molecular genetic testing and the future of clinical genomics. Nature Reviews. Genetics. 2013;**14**(6):415-26.

For peer review only

23. Chan IS, Ginsburg GS. Person-specificized medicine: progress and promise. Annual Review of Genomics and Human Genetics 2011;**29**(12):217-44.
24. Rapiti E, Pinaud K, Chappuis PO, et al. Opportunities for improving triple-negative breast cancer outcomes: results of a population-based study. Cancer Medicine. 2017;**6**(3):526-36.
25. Trainer AH, Lewis CR, Tucker K, et al. The role of BRCA mutation testing in determining breast cancer therapy. Nature reviews Clinical oncology. 2010;**7**(12):708-17.
26. Gray SW, O'Grady C, Karp L, et al. Risk information exposure and direct-to-consumer genetic testing for BRCA mutations among women with a person-specific or family history of breast or ovarian cancer. Cancer Epidemiology and Prevention Biomarkers. 2009;**18**(4):1303-11.
27. Annas GJ, Elias S. 23andMe and the FDA. New England Journal of Medicine 2014;**370**(11):985-88.
28. McGuire AL, Joffe S, Koenig BA, et al. Ethics and genomic incidental findings. Science 2013;**340**:1047-48.
29. Schuurman AG, van der Kolk DM, Verkerk MA, et al. Maximising the efficiency of clinical screening programmes: balancing predictive genetic testing with a right not to know. European Journal of Human Genetics 2015;**23**:1124-28.
30. Su Y, Borry P, Otte IC, et al. "It's our DNA, we deserve the right to test!" A content analysis of a petition for the right to access direct-to-consumer genetic testing. Personalized Medicine 2013;**10**(7):729-39.
31. Heshka JT, Palleschi C, Howley H, et al. A systematic review of perceived risks, psychological and behavioral impacts of genetic testing. Genetics in Medicine 2008;**10**(1):19-32.
32. Bosch N, Junyent N, Gadea N, et al. What factors may influence psychological well being at three months and one year post BRCA genetic result disclosure? The Breast 2012;**21**:755-60.
33. Ringwald J, Wochnowski C, Bosse K, et al. Psychological Distress, Anxiety, and Depression of Cancer-Affected BRCA1/2 Mutation Carriers: a Systematic Review. Journal of Genetic Counseling 2016;**25**:880-91.
34. Meiser B. Psychological impact of genetic testing for cancer susceptibility: An update of the literature. Psycho-Oncology 2005;**14**(12):1060-74.
35. Larouche G, Chiquette J, Pelletier S, et al. Do women change their breast cancer mammogram screening behaviour after BRCA1/2 testing? Familial Cancer 2016:1-6.
36. Beery TA, Williams JK. Risk reduction and health promotion behaviors following genetic testing for adult-onset disorders. Genetic Testing 2007;**11**(2):111-23.
37. Marteau TM, French DP, Griffin SJ, et al. Effects of communicating DNA-based disease risk estimates on risk-reducing behaviours. The Cochrane Library 2010.
38. Hollands GJ, French DP, Griffin SJ, et al. The impact of communicating genetic risks of disease on risk-reducing health behaviour: systematic review with meta-analysis. British Medical Journal 2016;**352**:i1102.
39. Biesecker BB, Ishibe N, Hadley DW, et al. Psychosocial factors predicting BRCA1/BRCA2 testing decisions in members of hereditary breast and ovarian cancer families. American Journal of Medical Genetics 2000;**93**(4):257-63.
40. Caulfield T, McGuire A, L. . Direct-to-consumer genetic testing: perceptions, problems, and policy responses. Annual Review of Medicine 2012;**63**:23-33.
41. Caulfield T, Ries NM, Ray PN, et al. Direct-to-consumer genetic testing: good, bad or benign? Clinical Genetics 2010;**77**(2):101-05.
42. Matloff E, Caplan A. Direct to confusion: lessons learned from marketing BRCA testing. The American Journal of Bioethics 2008;**8**(6):5-8.
43. George A, Kaye S, Banerjee S. Delivering widespread BRCA testing and PARP inhibition to patients with ovarian cancer. Nature Reviews Clinical Oncology. 2017;**14**(5):284-96.
44. Kentwell M, Dow E, Antill Y, et al. Mainstreaming cancer genetics: A model integrating germline BRCA testing into routine ovarian cancer clinics. Gynecologic Oncology. 2017;**145**(1):130-6.
45. George A, Riddell D, Seal S, et al. Implementing rapid, robust, cost-effective, patient-centred, routine genetic testing in ovarian cancer patients. Scientific reports. 2016; **13(**6):29506.

For peer review only

46. Plaskocinska I, Shipman H, Drummond J, et al. New paradigms for BRCA1/BRCA2 testing in women with ovarian cancer: results of the Genetic Testing in Epithelial Ovarian Cancer (GTEOC) study. Journal of Medical Genetics 2016.
47. Zilliacus EM, Meiser B, Lobb EA, et al. Are videoconferenced consultations as effective as face-to-face consultations for hereditary breast and ovarian cancer genetic counseling? Genetics in Medicine 2011;**13**(11):933-41.
48. Buchanan AH, Datta SK, Skinner CS, et al. Randomized trial of telegenetics vs. in-person cancer genetic counseling: Cost, patient satisfaction and attendance. Journal of Genetic Counseling 2015;**24**(6):961-70.
49. Meropol NJ, Daly MB, Vig HS, et al. Delivery of Internet-based cancer genetic counselling services to patients' homes: a feasibility study. Journal of Telemedicine and Telecare 2011;**17**:36–40.
50. Lerman C, Croyle RT, Tercyak KP, et al. Genetic testing: psychological aspects and implications. Journal of Consulting and Clinical Psychology 2002;**70**(3):784-97.
51. Ropka ME, Wenzel J, Phillips EK, et al. Uptake rates for breast cancer genetic testing: a systematic review. Cancer Epidemiology Biomarkers & Prevention 2006;**15**(5): 840-55.
52. Meisel SF, Side L, Fraser L, et al. Population-based, risk-stratified genetic testing for ovarian cancer risk: a focus group study. Public Health Genomics 2013;**16**(4):184-91.
53. Henneman L, Timmermans DR, Bouwman CM, et al. 'A low risk is still a risk': exploring women's attitudes towards genetic testing for breast cancer susceptibility in order to target disease prevention. Public Health Genomics 2011;**14**(4-5):238-47.
54. Ulrich CM, Kristal AR, White E, et al. Genetic testing for cancer risk: A population survey on attitudes and intention. Public Health Genomics 1998;**1**(14):213-22.
55. Henneman L, Vermeulen E, van El CG, et al. Public attitudes towards genetic testing revisited: comparing opinions between 2002 and 2010. European Journal of Human Genetics 2013;**21**(8):793-99.
56. Meisel SF, Pashayan N, Rahman B, et al. Adjusting the frequency of mammography screening on the basis of genetic risk: Attitudes among women in the UK. The Breast 2015;**24**(3):237-41.
57. Sanderson SC, Wardle J, Jarvis MJ, et al. Public interest in genetic testing for susceptibility to heart disease and cancer: a population-based survey in the UK. Preventive Medicine 2004;**39**(3):458-64.
58. Singer E. Public attitudes toward genetic testing. Population Research and Policy Review 1991;**10**(3):235-55.
59. Henneman L, Timmermans DRM, Wal GVD. Public Attitudes Toward Genetic Testing: Perceived Benefits and Objections. Genetic Testing 2006;**10**(2):139-45.
60. Gottfredson LS. Intelligence: is it the epidemiologists' elusive" fundamental cause" of social class inequalities in health? Journal of Personality and Social Psychology 2004;**86**(1).
61. Beeken RJ, Simon AE, von Wagner C, et al. Cancer fatalism: deterring early presentation and increasing social inequalities? Cancer Epidemiology Biomarkers & Prevention 2011;**20**(10):2127-31.
62. von Wagner C, Good A, Whitaker KL, et al. Psychosocial determinants of socioeconomic inequalities in cancer screening participation: a conceptual framework. Epidemiologic Reviews 2011;**33**(1):135-47.
63. Sanderson SC, Waller J, Jarvis MJ, et al. Awareness of lifestyle risk factors for cancer and heart disease among adults in the UK. Patient Education and Counseling 2009;**74**(2):221-27.
64. Kutner M, Greenberg E, Jin Y, et al. (2006) The Health Literacy of America's Adults: Results From the
2003 National Assessment of Adult Literacy (NCES 2006–483) U.S. Department of Education, Washington, DC: National Center for Education Statistics.
65. Lo SH, Waller J, Wardle J, et al. Comparing barriers to colorectal cancer screening with barriers to breast and cervical screening: a population-based survey of screening-age women in Great Britain. Journal of Medical Screening 2013;**20**(2):73-79.
66. Dent T, Jbilou J, Rafi I, et al. Stratified cancer screening: the practicalities of implementation. Public Health Genomics 2013;**16**(3):94-99.

For peer review only

67. Hall AE, Chowdhury S, Hallowell N, et al. Implementing risk-stratified screening for common cancers: a review of potential ethical, legal and social issues. Journal of Public Health 2014;**36**(2):285-91.
68. Lerman C, Daly M, Masny A, et al. Attitudes about genetic testing for breast-ovarian cancer susceptibility. Journal of Clinical Oncology 1994;**12**(4):843-50.
69. Bottorff JL, Ratner PA, Balneaves LG, et al. Women's interest in genetic testing for breast cancer risk the influence of sociodemographics and knowledge. Cancer Epidemiology Biomarkers & Prevention 2002;**11**(1):89-95.
70. Aro AR, Hakonen A, Hietala M, et al. Acceptance of genetic testing in a general population: age, education and gender differences. Patient Education and Counseling 1997;**32**(1):41-49.
71. Borzekowski DL, Guan Y, Smith KC, et al. The Angelina effect: immediate reach, grasp, and impact of going public. Genetics in Medicine 2013;**16**(7):516-21.
72. Kamenova K, Reshef A, Caulfield T. Angelina Jolie's faulty gene: newspaper coverage of a celebrity's preventive bilateral mastectomy in Canada, the United States, and the United Kingdom. Genetics in Medicine 2013;**16**:522-28.
73. Cameron LD, Reeve J. Risk perceptions, worry, and attitudes about genetic testing for breast cancer susceptibility. Psychology & Health 2006;**21**(2):211-30.
74. Lipkus IM, Klein WM, Skinner CS, et al. Breast cancer risk perceptions and breast cancer worry: What predicts what? Journal of Risk Research 2005;**8**(5):439-52.
75. Sheeran P, Webb TL. The Intention–Behavior Gap. Social and Personality Psychology Compass 2016;**10**(9):503-18.
76. Gilovich T, Griffin D, Kahneman D. *Heuristics and biases: The psychology of intuitive judgment*: Cambridge University Press, 2002.
77. Wilson TD, Gilbert DT. Affective forecasting. Advances in Experimental Social Psychology 2003;**35**:345-411.
78. Hann, KEJ, Freeman, M, Fraser, L, et al. Awareness, knowledge, perceptions, and attitudes towards genetic testing for cancer risk among ethnic minority groups: a systematic review. BMC Public Health 2017;17**:** 503.
79. Atkin, K, Ali N, Chu, CE. The politics of difference? Providing a cancer genetics service in a culturally and linguistically diverse society. Diversity in Health and Care 2009*;* 6**:** 149–157
80. Genetic Alliance UK. 2012. Identifying family risk of cancer: Why is this more difficult for ethnic minority communities and what would help? London.
81. Sanderson SC, Wardle J. Assosciations between anticipated reactions to genetic test results and interest in genetic testing: Will self-selection reduce the potential for harm? Genetic Testing 2008;**12**(1):59-66

Table 1: Sample demographic and personal characteristics (n=837)

	N (%)
**Gender**	
Female	837 (100.0)
**Age (mean, SD)**	45.9 (15.5)
**Age group**	
18-29 years	143 (17.1)
30-39 years	179 (21.4)
40-49 years	160 (19.1)
50-64	234 (28.0)
65-74 years	121 (14.5
**Ethnicity**	
White British	725 (86.6)
White Other	52 (6.3)
Mixed ethnicity	12 (1.4)
Black	11 (1.3)
South Asian	28 (3.3)
Other	9 (1.1)
**Education**	
No formal qualifications	126 (15.1)
Less than degree level qualification	478 (57.1)
Degree level (or equivalent)	233 (27.8)
**Marital status**	
Married/ Civil partnership/ Cohabiting	459 (54.8)
Single	177 (21.1)
Divorced/ Separated	140 (16.7)
Widowed	61 (7.3)
**Family history of cancer (mother, father, brother, sister)**	
Yes	305 (36.4)
No/don't know	532 (63.6)
**Perceived chances of getting ovarian cancer compared to other women of same age**	
Much higher/higher	64 (7.6)
About the same as others	590 (70.5)
Much lower/lower	183 (21.9)
**Perceived chances of getting breast cancer compared to other women of same age**	
Much higher/higher	83 (9.9)
About the same as others	616 (73.7)
Much lower/lower	137 (16.4)

For peer review only

For peer review only

Table 2. Univariate Analyses of anticipated reactions to risk disclosure of breast and ovarian cancer genetic risk

	**'I would try harder to have a healthy lifestyle'[1]**		**'It would give me more control over my life'[1]**		**'I would feel that it wasn't worth making lifestyle changes'[1]**		**'I would feel less free make choices in my l**	
	n (%) agree/ strongly agree	χ^2 (df) p-value	n (%) agree/ strongly agree	χ^2 (df) p-value	n (%) agree/ strongly agree	χ^2 (df) p-value	n (%) agree/ strongly agree	χ^2 (p-v
**otal (n = 837)**	611 (73.0)		452 (54.0)		119 (14.2)		143 (17.1)	
	248 (71.7)	0.52 (1)	199 (57.5)	2.92 (1)	54 (15.6)	0.93 (1)	55 (15.9)	0.5
	363 (73.9)	0.469	253 (51.5)	0.087	65 (13.2)	0.334	88 (17.9)	0.4
	567 (73.0)	0.004 (1)	415 (53.4)	1.52 (1)	111 (14.3)	0.04 (1)	**127 (16.3)**	**4.1**
**minority**	44 (73.3)	0.952	37 (61.7)	0.218	8 (13.3)	0.839	**16 (26.7)**	**0.0**
**n**								
**ity degree**	162 (69.5)	1.97 (1)	**112 (48.1)**	**4.57 (1)**	**21 (9.0)**	**7.17 (1)**	34 (14.6)	1.4
**niversity degree**	449 (74.3)	0.160	**340 (56.3)**	**0.032***	**98 (16.2)**	**0.007***	119 (18.0)	0.2
**tatus**								
**l/cohabiting**	343 (75.4)	2.87 (1)	**261 (57.4)**	**4.53 (1)**	61 (13.4)	0.53 (1)	67 (14.7)	**3.9**
**widowed/divorced**	268 (70.2)	0.090	**191 (50.0)**	**0.033***	58 (15.2)	0.463	76 (19.9)	**0.0**
**story of any cancer**								
**'t know**	385 (72.4)	0.29 (1)	**271 (50.9)**	**5.51 (1)**	73 (13.7)	0.29 (1)	98 (18.4)	1.8
	226 (74.1)	0.587	**181 (59.3)**	**0.019***	46 (15.1)	0.588	45 (14.8)	0.1
**l risk of OC (n = 847)**								
**ower than others**	40 (66.7)	6.92 (4)	30 (50.0)	4.08 (4)	11 (18.3)	1.26 (4)	**22 (36.7)**	**22**
**han others**	94 (76.4)	0.140	66 (53.7)	0.394	17 (13.8)	0.867	**25 (20.3)**	**<0**
**e as others**	427 (72.4)		314 (53.2)		83 (14.1)		**84 (14.2)**	
**than others**	48 (81.4)		39 (66.1)		7 (11.9)		**10 (16.9)**	
**igher than others**	2 (40.0)		3 (60.0)		1 (20.0)		**2 (40.0)**	
**l risk of BC (n = 847)**								
**ower than others**	16 (64.0)	3.58 (4)	8 (32.0)	5.17 (4)	6 (24.0)	3.46 (4)	**8 (32.0)**	**11**
**han others**	88 (78.6)	0.466	61 (54.5)	0.269	14 (12.5)	0.484	**27 (24.1)**	**0.0**
**e as others**	444 (72.0)		337 (54.6)		84 (13.6)		**92 (14.9)**	
**than others**	55 (75.3)		41 (56.2)		13 (17.8)		**13 (17.8)**	
**igher than others**	8 (80.0)		5 (50.0)		2 (20.0)		**3 (30.3)**	

[1]Each statement preceded by: If I knew my risk of breast and ovarian cancer, I would…; Abbreviations: BC = breast cancer, OC = ovarian cancer: * Significant at p <0.05

For peer review only

Table 3. Multivariate logistic regression of demographic and person-specific predictors of anticipated reactions to disclosure of genetic risk of breast and ovarian cancer

Variable (n = 837)	**‘I would try harder to have a healthy lifestyle’[1]**			**It would give me more control over my life’[1]**			**‘I would feel it wasn’t w[illegible] making lifestyle changes[illegible]**		
	OR	95% CI	p-value	OR	95% CI	p-value	OR	95% CI	[illegible]
**Age**									
**>50**	1			1			1		
**≤50**	1.86	0.84-1.66	0.321	0.80	0.60-1.11	0.205	0.99	0.64-1.53	[illegible]
**Ethnicity**									
**White**	1			1			1		
**Ethnic minority**	0.95	0.51-1.66	0.859	1.51	0.86-2.64	0.149	1.01	0.45-2.22	[illegible]
**Education**									
**University degree**	1			1			**1**		
**Below university degree**	1.35	0.96-1.91	0.081	1.39	1.01-1.91	0.039	**1.99**	**1.19-3.35**	[illegible]
**Marital status**									
**Single/widowed/divorced**	1			1			1		
**Married/cohabiting**	1.34	0.98-1.83	0.062	1.41	1.07-1.87	0.016	0.92	0.62-1.38	[illegible]
**Family history of any cancer**									
**No/don’t know**	1			1			1		
**Yes**	1.14	0.81-1.61	0.438	1.31	0.96-1.78	0.085	1.09	0.70-1.68	[illegible]
**Perceived risk of OC**									
**Much lower/lower than others**	1			1			1		
**The same as others**	1.15	0.68-1.95	0.588	0.95	0.58-1.55	0.848	0.90	0.47-1.72	[illegible]
**Higher/much higher than others**	1.41	0.64-3.15	0.392	1.86	0.90-3.84	0.092	0.54	0.19-1.51	[illegible]
**Perceived risk of BC**									
**Much lower/lower than others**	1			1			1		
**The same as others**	0.65	0.36-1.19	0.171	1.29	0.75-2.22	0.349	0.95	0.46-1.99	[illegible]
**Higher/much higher than others**	0.70	0.32-1.54	0.376	0.97	0.49-1.94	0.934	1.44	0.57-3.63	[illegible]

[1]Each statement was preceded by: If I knew my risk of breast and ovarian cancer…

Abbreviations: BC = breast cancer, OC = ovarian cancer

***** Significant after Bonferroni correction at p <0.012

For peer review only

Supplementary Table 1. Full set of responses for outcome variables

If I knew my risk of breast and ovarian cancer…	N (%)
**I would try harder to have a healthy lifestyle**	
Strongly disagree	33 (3.9)
Disagree	50 (6.0)
Neither agree/disagree	143 (17.1)
Agree	425 (50.8)
Strongly agree	186 (22.2)
**It would give me more control over my life**	
Strongly disagree	22 (2.6)
Disagree	135 (16.1)
Neither agree/disagree	228 (27.2)
Agree	387 (46.2)
Strongly agree	65 (7.8)
**I would feel it wasn't worth making lifestyle changes**	
Strongly disagree	169 (20.2)
Disagree	397 (47.4)
Neither agree/disagree	152 (18.2)
Agree	104 (12.4)
Strongly agree	15 (1.8)
**I would feel less free to make choices in my life**	
Strongly disagree	112 (13.4)
Disagree	402 (48.0)
Neither agree/disagree	180 (21.5)
Agree	129 (15.4)
Strongly agree	14 (1.7)
